# Growth characteristics of HCT116 xenografts lacking asparagine synthetase vary according to sex

**DOI:** 10.1186/s40246-024-00635-3

**Published:** 2024-06-17

**Authors:** Oladimeji Aladelokun, Lingeng Lu, Jie Zheng, Hong Yan, Abhishek Jain, Joanna Gibson, Sajid A. Khan, Caroline H. Johnson

**Affiliations:** 1 Department of Environmental Health Sciences, Yale School of Public Health, Yale University, New Haven, CT USA; 2 Department of Chronic Disease Epidemiology, Yale School of Public Health, Yale University, New Haven, CT 06510 USA; 3 Division of Surgical Oncology, Department of Surgery, Yale School of Medicine, New Haven, CT USA; 4 Department of Pathology, Yale University, New Haven, CT USA

**Keywords:** Metabolism, Colorectal cancer, *ASNS*, *GPER1*, Sex differences

## Abstract

**Background:**

Sex-related differences in colorectal (CRC) incidence and mortality are well-documented. However, the impact of sex on metabolic pathways that drive cancer growth is not well understood. High expression of asparagine synthetase (*ASNS*) is associated with inferior survival for female CRC patients only. Here, we used a CRISPR/Cas9 technology to generate HCT116 *ASNS*^−/−^ and HCT 116 *ASNS*^+/+^ cancer cell lines. We examine the effects of *ASNS* deletion on tumor growth and the subsequent rewiring of metabolic pathways in male and female Rag2/IL2RG mice.

**Results:**

*ASNS* loss reduces cancer burden in male and female tumor-bearing mice (40% reduction, q < 0.05), triggers metabolic reprogramming including gluconeogenesis, but confers a survival improvement (30 days median survival, q < 0.05) in female tumor-bearing mice alone. Transcriptomic analyses revealed upregulation of G-protein coupled estrogen receptor (*GPER1*) in tumors from male and female mice with HCT116 *ASNS*^−/−^ xenograft. Estradiol activates *GPER1* in vitro in the presence of *ASNS* and suppresses tumor growth.

**Conclusions:**

Our study indicates that inferior survival for female CRC patients with high *ASNS* may be due to metabolic reprogramming that sustains tumor growth. These findings have translational relevance as *ASNS*/*GPER1* signaling could be a future therapeutic target to improve the survival of female CRC patients.

**Supplementary Information:**

The online version contains supplementary material available at 10.1186/s40246-024-00635-3.

## Background

Colon and rectal cancers are the third most common cause of cancer deaths in women and men in the United States [1, 2]. Sex-related differences in CRC incidence and mortality have been previously documented and have shown that even though males have a higher incidence and mortality, females still have a high incidence rate of 34.0 per 100,000 population, with more frequent lesions on the right-side of the colon; an area of the colorectum that confers a poorer clinical outcome for those with tumors [3, 4]. Interestingly, right-sided tumors are more likely to carry a *KRAS* mutation, and *KRAS* mutations are more frequently observed in tumors from female CRC patients [5].

Mechanistically, KRAS has major implications on tumor metabolism. Evidence shows that KRAS plays a role in rewiring metabolic pathways and regulating estrogen receptor signaling, highlighting the relevance of these factors when considering treatment for cancers in females [6, 7]. Thus, ongoing efforts are now focused on targeting alternative pathways that are activated in mutant *KRAS* cells, to circumvent drug resistance [8]. Moreover, oncogenic *KRAS* signaling can promote metabolic reprogramming through stimulation of glucose uptake and diversion of nutrients towards anabolism, a phenomenon termed the Warburg effect. Cancer cells increase uptake of nutrients such as glucose and glutamine, which are used as cellular building blocks that enhance tumor growth. However, the mechanism by which oncogenic *KRAS* coordinates metabolic reprogramming to promote tumor growth remains an area of intense investigation [7, 9]. Studies have shown that nutrient supply is an important determinant of KRAS regulation of cancer. Under nutrient deprivation, activation of downstream intracellular molecules such as activating transcription factor 4 (ATF4) can stimulate the synthesis of non-essential amino acids (NEAA) such as glutamine. This finding is important especially with respect to glutamine metabolism, where *KRAS*-mutant cancer cells adapt to glutamine withdrawal by increasing asparagine biosynthesis via asparagine synthetase (*ASNS*), which allows cancer cells to survive [10]. Indeed, indirect mechanisms of glutamine reliance are related to induction of ATF4 that can influence *ASNS* activity. Furthermore, *ASNS* facilitates the synthesis of asparagine in a glutamine and ATP-dependent manner [9, 11]. This cancer cell dependence on glutamine can also lead to replenishing of important TCA cycle intermediates for rapid cell growth [12]. Asparagine in particular, has been shown to play a critical role in regulating cell proliferation and promoting tumor metastasis, and is thus an important amino acid during cancer progression [9, 13].

We have previously examined *ASNS* expression levels in colon cancer patients from The Cancer Genome Atlas (TGCA) colon adenocarcinoma (COAD) cohort and revealed that female patients with high *ASNS* expression had an inferior survival compared to females with low-medium *ASNS* expression, whereas these associations between *ASNS* and outcomes not exist in male patients, suggesting sex-specificity for CRC outcomes based on *ASNS* [14–16]. Using metabolomics data acquired from patient tumors, we showed that high asparagine compared to low asparagine levels are associated with poorer survival and recurrence-free survival in female patients only [15]. Additional analysis of TCGA COAD data by stage showed that female patients only with stage III-IV CRC, with high versus low *ASNS* expression had a poor overall survival (*p* < 0.05) [16]. Whereas there were no significant difference (*p* > 0.05) in overall survival found for female patients with early-stage tumors (stage I-II) [16]. Together, this highlights the importance of *ASNS* in cancer cells undergoing metabolic stress. Thus, we hypothesize that ASNS may serve as key molecular factor for understanding sex-biased differences in cancer incidence and survival [7, 17].

Given these findings, we aimed to address two important questions in this study. First, how does dysregulated *ASNS* signaling alter tissue homeostasis and induce tumor progression? Using an in vivo model of human colon tumorigenesis (isogenic HCT116 *ASNS*^+*/*+^ and HCT116 *ASNS*^*−/−*^ cell line-derived xenografts which express mutant *KRAS*^G13D^ endogenously), biochemical and metabolic factors identified in this study provide novel insights into the mechanisms of metabolic rewiring, cell proliferation and tumor growth. Second, what factors are responsible for the sex-specific differences in CRC using this model of human tumorigenesis that potentiates *ASNS* signaling? We identify GPER1 signaling as a potential therapeutic target that can be exploited for CRC treatment [18, 19]. We address a void in the scientific literature by showing a relationship between *GPER1* and *ASNS* signaling. We speculate that there could be hormonal effects on *ASNS* mutant clones. Our study proposes new hypotheses for the mechanisms of sex-specific influences of *ASNS*-driven asparagine metabolism using a mouse xenograft model of CRC.

## Methods

### Cell culture

Human CRC HCT116 cell lines were obtained from American Type Culture Collection (ATCC) (Manassas, VA, USA). CRISPR-Cas9 editing was performed to generate HCT116 *ASNS* wild-type (*ASNS*^+/+^) and HCT 116 *ASNS* knock-out (*ASNS*^−/−^) (Synthego Corp, Redwood City, (CA, USA)). HCT116 *ASNS*^−/−^ cell lines were generated using a CRISPR-mediated gene knockout. The wildtype pool was mock transfected with Cas9 which served as control cell line for the downstream experiment. *ASNS*^−/−^ status was confirmed by western blot analysis. Cells were maintained in Dulbecco’s Modified Eagle Medium (DMEM) (Thermo Fisher Scientific, Waltham, MA, USA) with 10% fetal bovine serum (Life Technologies, Carlsbad, CA, USA) and 1% penicillin/streptomycin (Life Technologies, Carlsbad, CA, USA) and 4 mM L-Glutamine (Life Technologies, Carlsbad, CA, USA). For the L-asparaginase (L-Asp) experiment, both HCT116 *ASNS*^+/+^ and *ASNS*^−/−^ cells were grown in RPMI-1640 culture medium (Thermo Fisher Scientific, Waltham, MA, USA) with sufficient asparagine and supplemented with 4 mM glutamine to scale up before exposure to L-Asp. For the metabolomics analysis, HCT116 *ASNS*^+/+^ and HCT116 *ASNS*^−/−^ cells were cultured in RPMI-1640 and supplemented with 4 mM glutamine. Cells were cultured at 37 °C and under 5% CO_2_. All experiments were performed at 90% cell confluency with no more than ten [10] cell passages. After three [3] passage rounds, cells were screened for biological contaminants at the Molecular Diagnostics laboratory (Yale University School of Medicine) and were declared to be pathogen-free. Amino acid composition of asparagine-depleted medium is presented in Supplementary Table S3.

### Thawing process

Cells were stored in LN_2_ and placed in a temperature-controlled water bath for 2 min 50 s equilibrated to 37 °C according to a previously reported standard method for thawing conventional cryovials [20]

### Formation of 3D spheres using hanging- drops

Adherent cell cultures were grown to at least 90% confluence before rinsing off monolayers with PBS. Cells were trypsinized using 0.05% trypsin with EDTA and incubated at 37 °C until cells detach. Trypsinization was stopped by adding 2mls of complete medium before transfer to a 15 ml conical tube. Cells were counted using a hemacytometer. 20 μl droplets were deposited onto a 100 mm tissue culture plate lid ensuring that each droplet was placed sufficiently apart. Lid was inverted onto a PBS-filled bottom chamber and incubated at 37 °C/5% CO_2_ and 95% humidity. At least 10 droplets/treatment group were deposited for the medium-asparagine depletion experiment. For the L-asparaginase experiment, at least 8 droplets/treatment condition were deposited onto the culture plate. Sizes of formed spheroids were evaluated using a light microscope.

### Animal experiments

Rag2/IL2RG double knockout (R2G2) immunocompromised mice, having reduced functions of T, B and Natural Killer (NK) cells, were obtained from Charles River Laboratories (Wilmington, MA, USA) at 4 weeks old. Animals were held in the Yale West Campus Animal husbandry unit until ready for xenograft (IACUC #2021-20218).

#### In vivo* tumorigenesis*

HCT116 *ASNS*^+/+^ and HCT116 *ASNS*^−/−^ were grown in complete medium and harvested for in vivo studies. 1 × 10^7^ cells were implanted subcutaneously (s.c) into the flanks of R2G2 mice. Tumor sizes were obtained in two dimensions by measuring with calipers every three days. Tumor volumes were calculated using the formular V = (L × W^2^)/2, where L is length and W represents width.

In study 1, ten female R2G2 mice were used for the tumorigenicity experiment. HCT116 *ASNS*^−/−^ and HCT116 *ASNS*^+/+^ were implanted subcutaneously (s.c) into the flank of each mouse (n = 5/group). Animal survival was evaluated from the day of cell line implantation until the mice became moribund according to predetermined criteria that includes a tumor volume of ≥ 1 cm^3^ [21, 22]. Animal husbandry and experimental procedures were carried out under approval from Yale University Institutional Animal Care and Use Committee (IACUC #2021-20218). All the animals were housed in specific-pathogen free (SPF) conditions and automatically supplied rodent chow (Teklad #2018S, Envigo, Indianapolis, IN, USA) and water ad libitum.

Once tumorigenicity was confirmed, 40 R2G2 mice (n = 20 female, n = 20 male) were divided into four groups for a tumor-specific survival experiment (Study 2); of note the tumor metabolomics analyses were performed in this mice cohort. In the survival study the following groups were constructed; group I included R2G2 male mice that were injected with HCT116 *ASNS*^+/+^ cell lines; group II included R2G2 male mice that were injected with HCT116 *ASNS*^−/−^ cell lines; group III, included R2G2 female mice that were injected with HCT116 *ASNS*^+/+^ cell lines; group IV included R2G2 female mice that were injected with HCT116 *ASNS*^−/−^ cell lines. For studies 1–2, each mouse received 1 × 10^7^ cells that were implanted subcutaneously (s.c) into the flank.

For study 1, mice were euthanized at day 20 as tumors in the HCT116 *ASNS*^+/+^ group reached 1000 mm^3^ which is the maximum tumor volume allowed by our protocol. At the time of euthanasia for both studies, a small section of tumor mass was examined grossly, fixed in 10% neutral buffered formalin and was used in making formalin-fixed paraffin-embedded (FFPE) blocks; while a second section of tumor mass was flashed frozen in liquid nitrogen (LN2).

### Histopathology

FFPE tissues from the tumorigenicity and survival studies were sectioned at 5 µm, stained with hematoxylin and eosin for histologic examination as previously described [23]. Unstained slides were subjected to immunohistochemical analysis. Briefly tissue sections were deparaffinized, antigen retrieved in 10 mM sodium citrate and incubated with 3% hydrogen peroxide for 20 min at room temperature (RT). Sections were blocked with 10% goat serum and incubated overnight at 4 °C with primary antibodies: cyclin D1 (CME432, Biocare Medical, Pacheco, CA, USA), phospho-histone H3 (#9701, Cell Signaling Technology, Danvers, MA, USA), F4/80 (#70,076, Cell Signaling Technology, Danvers, MA, USA) and Ki67 (CRM325, Biocare Medical, Pacheco, CA, US). Sections were then incubated with secondary antibody for 30 min at room temperature, followed by signal detection using 3,3’-diaminobenzidine (DAB) solution (#SK-4105, Vector Laboratories Inc., Newark, CA, USA). Tissues were counterstained with hematoxylin. A pathologist with expertise in gastrointestinal pathology examined representative sections. 

### Mitotic activity index

The mitotic activity index of the tumor cells was defined as either the number of tumor cells with mitotic figures (representing dividing cells) or Ki67-positive tumor cells, divided by total number of tumor cells. For quantitative evaluations, a minimum of ten fields (40x) was selected and mitotic activity index was calculated as the average number of pHH3^+^ cells per field. At least 3 representative samples were randomly selected from each group in study 1 and 2.

### Drugs and chemicals

G1(1-((3aR,4S,9bS)-rel-4-(6-Bromobenzo[d][1,3]dioxol-5-yl)-3a,4,5,9b-tetrahydro-3H-cyclopenta[c**]** quinolin-8-yl)ethanone, 98%, a GPER1 agonist, was purchased from Ambeed, #A287698 (CAS: 881,639–98-1, Arlington Heights, IL, USA). L-asparaginase (#ENZ-287-500 IU) was purchased from Peptide International Inc (Louisville, KY, USA).

### Immunoblot

To confirm the efficiency of the CRISPR-Cas9 Knock out system, we assessed the relative protein expression of ASNS in the HCT116 *ASNS*^+/+^ and *ASNS*^−/−^ mutant cell lines using western blot. We also confirmed the expression of GPER1 in HCT116 *ASNS*^+/+^ cells after pharmacological modulation with estradiol and G1 (a known GPER1 agonist). Proteins were extracted by homogenization using 1 × RIPA buffer with protease inhibitor (#5,892,970,001, Roche, Basel, Switzerland). The homogenates were centrifuged at ≥ 10,000 g at 4 °C, and the supernatants were heated for 10 min in 1 × sodium dodecyl sulfate (SDS) sample buffer for protein denaturation. After a brief centrifugation, 30 μg of protein was resolved on SDS–polyacrylamide gels and transferred to nitrocellulose membranes by voltage gradient transfer. The membranes were incubated in 3% bovine serum albumin in TBST buffer for one hour to block non-specific binding; then incubated with primary antibodies (anti-ASNS: #sc-365809, Santa Cruz biotechnology, Dallas, TX, USA; anti-GPER1: #ab260033, Abcam Biotechnology, Cambridge, UK; anti-GAPDH #ab181602, Abcam Biotechnology, Cambridge, UK) containing 3% bovine serum albumin in TBST at 4 °C overnight. Membranes were then washed and incubated with secondary antibodies coupled to horseradish peroxidase for 1 h. The bands were detected using a chemiluminescence reagent kit (#PI32106, Thermofisher Scientific, Waltham, MA, USA). Quantification of band intensity was performed on all stained blots using Image J processing software. For GPER1 antibody which detect an additional band of GPER heterodimers (above the 50 kDa ladder), the lower band was selected and normalized to house keeping protein (GAPDH). Inverted background value was subtracted from all membranes and expression is reported as the net ratio of either ASNS or GPER1 protein to GAPDH.

### Tissue microarray

Corresponding paraffin tissue blocks of R2G2 mice tumors (HCT116 *ASNS*^+/+^ female, HCT116 *ASNS*^−/−^ female, HCT116 *ASNS*^+/+^ male and HCT116 *ASNS*^−/−^ male; n = 5 mice tumors/group) were retrieved and transferred into a recipient block or tissue microarray (TMA). TMA cores were punched out using a 0.6 mm diameter tool for up to 2 punches per tumor block and transferred onto digital slides. H&E slides from selected mouse tumors were also retrieved. Blocks and slides were compared in an identical manner using an annotated grid. Immunohistochemistry of Ki67, cyclin D1 and pHH3 were conducted on TMA slides. Ki67, cyclin D1 and pHH3-positive cells were counted using Image J processing software.

### RNA-sequencing

RNA-seq was performed on female mice tumors from the tumorigenicity study (n = 4 HCT116 *ASNS*^+/+^ and n = 4 HCT116 *ASNS*^−/−^). Libraries were prepared with total RNA using the Total RNA TruSeq mRNA Stranded Library Prep Kit (Illumina, San Diego, CA, USA). Briefly, mRNA pulldown was performed using an oligodT primer attached to magnetic beads. The libraries were quantified using the Qubit dsDNA HS assay kit (Thermofisher Scientific, Waltham, MA, USA) and an Agilent 2100 Bioanalyzer. Pooled library was sequenced on an efficient ultra-high-throughput sequencer using the NovaSeq 6000, an instrument with a patterned flow cell technology which provides the highest throughput across multiple applications (Illumina) according to the manufacturer’s protocol. A detailed sequencing procedure is described in the supplementary material and method section.

### Quantitative real-time PCR

Total RNA was isolated from tumors of HCT116 xenografts using the RNA isolation kit (Rneasy Mini Kit, QIAGEN, Hilden, Germany). RNA quality control was determined as described above for RNA sequencing. Extracted RNA was reversed transcribed to generate complementary DNA (cDNA) using the iScript cDNA synthesis kit (Bio-Rad, Hercules, CA, USA) following the manufacturer’s instructions. cDNA was diluted in nuclease-free water before it was utilized for gene expression by qRT-PCR. Quantitative real-time PCR analysis was performed on technical duplicates of cDNA derived from Study 1: HCT116 *ASNS*^+/+^ female group (n = 4) and *ASNS*^−/−^ female group (n = 3) and Study 2: HCT116 *ASNS*^+/+^ male (n = 8), HCT116 *ASNS*^−/−^ male (n = 8), HCT116 *ASNS*^+/+^ female (n = 10) and HCT116 *ASNS*^*−/−*^ female (n = 8–9) groups, using iTaq SYBR Green (Bio-Rad, Hercules, CA, USA), and predesigned validated primers on CFX96 Touch Real-Time PCR Detection System (Bio-Rad, Hercules, CA, USA). Primer sequences for qRT-PCR are reported below:

*hGPER1* fw 5′-ACAAACCCAACCCAAACCAC-3′

*hGPER1* rev 5′-CACCGTGCAGCTTTCAAGAT-3′

*hASNS* fw 5′- GACTTCACCTGATAAAAGGCAGC-3′

*hASNS* rev 5′- TTGAAGCACTCCGCGACTCC-3′

*hGAPDH* fw 5′- GCAGGGGGGAGCCAAAAGGG-3′

*hGAPDH* rev 5′- TGCCAGCCCCAGCGTCAAAG-3′

### Untargeted mass spectrometry

#### Tumor metabolite extraction

Tumor metabolomics was performed on tumors from the survival study. 40 tumors, at 50 mg tumor tissue was homogenized in 500 μL UPLC-grade H_2_O using a Precellys Evolution cryo-homogenizer (Bertin Corp. Rockville, MD, USA). 2 mL lysis tubes containing 1.4 mm ceramic (zirconium oxide) beads were utilized for the homogenization step. Prior to homogenization, tissue samples were cut on a pre-chilled cutting board kept on dry ice. During homogenization, samples were kept on ice and each sample was processed at 6,000 rpm for 20 s at 6 cycles with 5 s intervals. Cryo-homogenizer was kept chilled using dry ice. 100 μL of homogenized solution was added to 1.5 mL microcentrifuge tubes (Eppendorf, Framingham, MA, USA) for subsequent metabolite extraction. A volume of ice cold 400 μL methanol:acetonitrile (1:1, v/v) was added to each sample as the extraction solvent before sonication for 10 min. Samples were incubated for 2 h at − 20 °C, followed by centrifugation at 13,000 rpm (15,000 g) and 4 °C for 15 min. To precipitate proteins and particulates, the resulting supernatant was removed and evaporated to dryness for 12 h using a vacuum concentrator (Thermo Fisher Scientific, Waltham, MA, USA). The dry extracts were then reconstituted in 100 µL of ACN:H_2_O (1:1, v/v), sonicated for 10 min, and centrifuged at 13,000 rpm (15,000 g) and 4 °C for 15 min to remove insoluble debris. The supernatant was transferred to UPLC autosampler vials (Thermo Fisher Scientific, Waltham, MA, USA). A pooled quality control sample was prepared by mixing 2 μL of extracted solution from each sample into a UPLC autosampler vial. All the vials were capped and stored at − 80 °C prior to UPLC-MS analysis.

#### UPLC-MS-based metabolomics analysis

Hydrophilic interaction chromatography (HILIC) and reverse-phase liquid chromatography (RPLC)-mass spectrometry (MS) approaches were used for comprehensive analysis of the tissue metabolome and have been described previously [14]. Briefly a UPLC system (H-Class ACQUITY, Waters Corporation, MA, United States) coupled to a quadrupole time-of flight (QTOF) mass spectrometer (Xevo G2-XS QTOF, Waters Corporation, Milford, MA, United States) was used for MS data acquisition. A Waters ACQUITY UPLC BEH Amide column and Waters ACQUITY UPLC BEH C18 column were used for the UPLC-based separation of metabolites. Column temperature was kept at 25 °C for HILIC-MS analysis and 30 °C for RPLC-MS analysis. Sample injection volume was 1 μL. The solvent flow rate was 0.5 mL/min. For HILIC-MS analysis, mobile phase A was 25 mM NH4OH and 25 mM NH4OAc in water, while the mobile phase B was ACN for both electrospray ionization (ESI) positive and negative mode, respectively. The linear gradient was set as follows: 0–0.5 min: 95% B; 0.5–7 min: 95% B to 65% B; 7–8 min: 65% B to 40% B; 8–9 min: 40% B; 9–9.1 min: 40% B to 95% B; 9.1–12 min: 95% B. For RPLC-MS analysis, the mobile phases A was 0.1% formic acid in H2O, while the mobile phases B was 0.1% formic acid in ACN, respectively for both ESI+ and ESI− . The linear gradient was set as follows: 0–1 min: 1% B; 1–8 min: 1% B to 100% B; 8–10 min: 100% B; 10–10.1 min: 100% B to 1% B; 10.1–12 min: 1% B. Pooled samples were analyzed at specified injections interval during the UPLC-MS analysis to monitor the stability of the data acquisition and used for subsequent data normalization. QTOF-MS scan data (300 ms/scan; mass scan range 50–1000 Da) was initially acquired for each biological sample for metabolite quantification. Then, both DDA (data-dependent acquisition) data (QTOF MS scan time: 50 ms/scan, MSMS scan time 50 ms/scan, collision energy 20 eV, top 5 most intense ions were selected for fragmentation, exclude former target ions (4 s after 2 occurrences)) and MS^2^ data (low energy scan: 200 ms/scan, collision energy 6 eV; high energy scan: 100 ms/scan, collision energy 20 eV, mass scan range 25–1000 Da) were acquired for QC samples to enable metabolite identification. ESI source parameters on the Xevo GS-XS QTOF were set as following: capillary voltage 1.8 kV, sampling cone 40 V, source temperature 50 °C, desolvation temperature 550 °C, cone gas flow 40 L/Hr, desolvation gas flow 900 L/Hr.

### UPLS-MS data processing

Raw MS data (.raw) were converted to mzML files using ProteoWizard MSConvert (http://proteowizard.sourceforge.net/). The files were then processed in R (version 4.2.1) using the XCMS package for feature detection, retention time correction and alignment. The XCMS processing parameters were optimized and set as follows: mass accuracy for peak detection = 25 ppm; peak width c = (2, 30) ; snthresh = 6; bw = 10; mzwid = 0.015; minfrac = 0.5. The CAMERA package was used for subsequent peak annotation. The resulting data were normalized using the support vector regression algorithm in R to remove unwanted system error that occurred among intra- and inter- batches. Initial metabolite identification was performed using the MetID (metabolomics standard initiative level 1). Metabolites were further identified by matching retention time with an in-house metabolite standard library.

#### Quantification and statistical analysis

Kaplan–Meier survival curves were compared by log-rank test. Mantel-Cox Chi square test of association was used to determine significance. For the tumor kinetics in study 2, two-way ANOVA with mixed-effects model was used to accommodate the maximum tumor volume reached in several of the HCT116 *ASNS*^+/+^ male mice starting day 9 post tumor implantation. Multiple comparisons were adjusted using Benjamini and Hochberg’s FDR method. Tumor volume for each time point was considered significant difference if q value is less than 0.05. For differential gene expression (DEG), comparison was determined using a negative binomial distribution test. Individual dot plots of RPKM are represented as the mean ± standard error of mean (SEM) with at least n = 4 per group and *p* values determined by unpaired student’s *t* test. For gene expression assay in study 2, statistical significance was determined using, two-way ANOVA. For all the qRT-PCR reactions, relative quantification of mRNA levels by the ΔΔCT method was performed after normalization of total cDNA to endogenous GAPDH. For metabolomics analysis, normalized metabolite abundances were uploaded in Metaboanalyst and Welches t-test was applied to compare metabolite abundances between sample groups. *p* values were adjusted for multiple testing with Benjamini-Hochberg-based FDR. For the immunohistochemistry analysis, individual dot plots represent mean ± SEM of at least 3 representative samples from each group of HCT116 *ASNS* xenografts.

#### Bioinformatics analysis

Functional enrichment analysis of differentially expressed genes by hierarchical clusters was performed using the Gene Set Enrichment Analysis (GSEA), a tool that offers a robust analytic technique for molecular profiling data [24]. Top canonical pathways and cellular functions involving differentially expressed transcripts (gene set > 15) using normalized expression count and weighted enrichment statistic. DEGs used in pathway analysis were determined between HCT116 *ASNS*^−/−^ and HCT116 *ASNS*^+/+^ by using a filtering criterion: fold change (FC) > 2.0 and *q* < 0.05. Ingenuity Pathway Analysis (IPA) (QIAGEN Redwood City, USA) was used to identify specific pathways and functions across sample groups. An unadjusted p-value was used to determine gene set enrichment as our hypothesis is that a coordinated change to a broad number of genes can aid in the prediction of changes to the greater signaling network. Top canonical pathways involving differentially altered metabolites (using metabolites with adjusted p-value < 0.05) were calculated based on the Fisher’s right-tailed exact test. Metabolomics profiling was conducted using IPA and violin plots were generated on Graphpad Prism Software (version 10).

## Results

### Loss of *ASNS* prevents compact spheroid formation in vitro and inhibits tumorigenesis in vivo

**Fig. 1:**
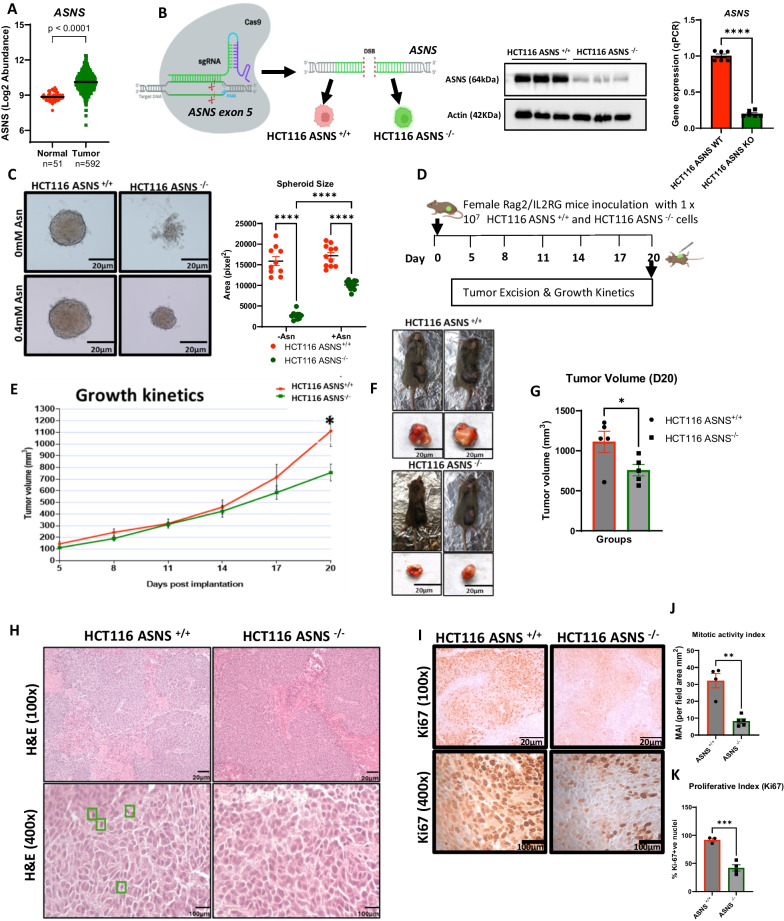
Loss of asparagine synthetase (*ASNS*) prevents compact spheroid formation in asparagine (Asn) adjusted media and inhibits tumorigenesis in vivo*. ***A***. ASNS* transcript levels in human normal-adjacent colon tissues (n = 51) and colorectal adenocarcinoma tissues (n = 591) from TCGA (COAD) database. **B.** Selective targeting of *ASNS* exon 5 in HCT116 cancer cell line. *ASNS*^−/−^ cell line was generated via CRISPR-Cas9 mediated deletion of exon 5. Mock-transfection of HCT116 wild type *ASNS* cells with Cas9. Western blot analysis confirmed the knock-out of ASNS showing reduced protein expression. qPCR revealed a significant reduced number of *ASNS* transcript in the CRISPR-Cas9 edited cell line compared to the *ASNS*^+/+^, thus confirming a knock-out efficiency. **C.** HCT116 *ASNS*^+*/*+^ and HCT116 *ASNS*^*−/−*^ cell lines were cultured in DMEM and RPMI media. Representative images of spheroids derived from *ASNS*^+*/*+^ pool and a CRISPR-Cas9 edited clones grown under asparagine depleted conditions (0 mM Asn) and Asn supplemented conditions (4 mM Asn). Spheroid image captured 7 days after seeding using 4× objective lens of an inverted microscope. *Note* the structural differences and”fuzziness” around the edges in the *ASNS*^+/+^ cell lines. **D** Experimental design of tumorigenicity experiment using Rag2/IL2RG (R2G2) immunocompromised mice, *n* = 5 mice per group. Female R2G2 immunodeficient mice were inoculated with ten million cells each of CRISPR- edited *ASNS*^-/-^ and the control *ASNS*^+/+^ lines. Day 0 represents the day when cell inoculation was performed. **E** Tumor kinetics reveal a significant difference (*p* < 0.05) in tumor volume of HCT116 *ASNS*^−/−^ mice compared to HCT116 *ASNS*^+/+^ mice at day 20 post-implantation. **F** Representative images of mice tumors from each group. *Note* that tumor size was relatively smaller in the HCT116 *ASNS*^−/−^ mice compared to the HCT116 *ASNS*^+/+^ mice. **G** Average tumor volume at day 20 post implantation. **H** Hematoxylin and eosin staining of tumors showing mitotic activity index in the HCT116 *ASNS*^+/+^ and HCT116 *ASNS*^−/−^ tumor-bearing female mice. **I** Reduced Ki67 immunostaining in the HCT116 *ASNS*^−/−^ tumors compared to HCT116 *ASNS*^+/+^ tumors. **J** Quantification of mitotic activity index in the HCT116 *ASNS*^+/+^ and HCT116 *ASNS*^−/−^ tumors. **K** Proliferative index for HCT116 *ASNS*^+/+^ and HCT116 *ASNS*^−/−^ indicated by Ki67 reactivity. Green box indicates mitotic figures. Representative hematoxylin and eosin staining of tumors derived from HCT116 *ASNS*^+/+^ and HCT116 *ASNS*^−/−^ mice under 100 × total magnification. Mitotic figures (MFs) in the HCT116 ASNS^+/+^ and HCT116 *ASNS*^−/−^ tumors under 400× total magnification. Extraction of *ASNS* expression dataset from TCGA database was performed using R studio and individual scattered dot plot represents expression Log 2 abundance of *ASNS*. Individual scattered plots for gene expression analysis of HCT116 *ASNS*^+/+^ and HCT116 *ASNS*^−/−^ cells (Fig. 1B), are n = 6 replicates per genotype. Individual scattered plots for spheroid experiments are mean ± SEM of at least 10 spheroids/group. Statistical significance indicated by *p < 0.05, ***p < 0.001 and ****p < 0.0001 using two-tailed Student’s *t*- test

Analysis of data from TCGA COAD showed that *ASNS* mRNA expression in human colorectal adenocarcinoma is higher than in normal-adjacent colon tissues **(**Fig. 1A**).** To gain insight into the roles of ASNS in CRC cell proliferation, we used isogenic cells that differ only by *ASNS* expression; human HCT116 CRC cells are wild-type for *ASNS*, and carry a *KRAS* (G13D) mutation [25, 26]. Using a CRISPR-Cas9 system, HCT116 *ASNS*^−/−^ clones were derived via homozygous knockout of *ASNS* by targeting exon 5 of the gene which encodes the active site of the protein. We confirmed *ASNS* deletion in the knockout cell lines by western blot and RT-qPCR (Fig. 1B).

We next investigated the impact of *ASNS* and the presence of asparagine on the formation and growth of multicellular spheroids; a useful surrogate for modeling cellular dynamics, differentiation and architecture of tumor in a 3D culture system [27] (Fig. 1C). HCT116 *ASNS*^−/−^ cell lines did not form a compact spheroid compared to the HCT116 *ASNS*^+/+^ cells under an asparagine-depleted media condition (0 mM asparagine). HCT116 *ASNS*^−/−^ cells were loosely attached to the cell culture dish and formed irregular clusters (Fig. 1C). However, the spheroid size of the HCT116 *ASNS*^+/+^ and HCT116 *ASNS*^−/−^ cells increased upon introduction of asparagine to the media (0.4 mM asparagine) (Fig. 1C). We observed fuzziness in sub-populations of HCT116 *ASNS*^+/+^ cells cultured under both asparagine deplete and asparagine replete conditions which enhanced their expansion, suggesting an ability to migrate from the parental spheres [27]. However, spheroid size was significantly smaller (2.1-fold, *p* < 0.001) in the HCT116 *ASNS*^−/−^ cell lines compared to the HCT116 *ASNS*^+/+^ group seven days after cell seeding under an asparagine-sufficient condition (Fig. 1C), thus highlighting the role of *ASNS* in initiating CRC cell proliferation and growth using a 3D culture system.

To confirm the importance of circulating asparagine on cell growth, we employed the use of L-asparaginase (L-Asp), a drug proven to have therapeutic efficacy against acute lymphoid leukemia (ALL) by reducing extracellular (i.e. circulating) asparagine through its conversion to aspartate [15]. L-Asp was added to the media of the HCT116 *ASNS*^−/−^ and HCT116 *ASNS*^+/+^ cell lines at multiple concentrations (0.5 IU/mL, 2 IU/mL and 4 IU/mL) (Supplementary Fig. 1A). RPMI media was used which contained sufficient levels of asparagine to increase the initial starting numbers of cells. We observed a dose-dependent reduction of formed spheroids caused by L-Asp at the three tested concentrations. Treatment of HCT116 *ASNS*^+/+^ cells with L-Asp at a low dose (0.5 IU/mL) caused a 33.9% reduction (*p* < 0.001) in spheroid size compared to untreated HCT116 *ASNS*^+/+^ cells (Supplementary Fig. 1B). Medium dose L-Asp (2 IU/mL) caused a 73.2% reduction (*p* < 0.001) in spheroid size compared to the untreated control group. Similarly, treatment with a high dose L-Asp (4 IU/mL) caused a pronounced 88.6% reduction (*p* < 0.001) in spheroid size of the HCT116 *ASNS*^+/+^ cells compared to the untreated control group Supplementary Fig. 1B). In HCT116 *ASNS*^−/−^ cells, 0.5 IU/mL L-Asp treatment caused a dramatic reduction (56.6%, *p* < 0.0001) in spheroid size compared to untreated cells. Medium dose (2 IU/mL) L-Asp caused a significant 88.3% reduction (*p* < 0.0001) in spheroid size of the HCT116 *ASNS*^−/−^ cells. A high concentration (4 IU/mL) of L-Asp drastically impacted (100% reduction, *p* < 0.0001) spheroid formation of the HCT116 *ASNS*^−/−^ cells. Quantification of these cells showed that the L-Asp drastically reduced spheroid formation and size to a larger degree (*p* < 0.0001) in the HCT116 *ASNS*^−/−^ lines compared to the wild-type counterpart (Supplementary Fig. 1B). To validate the contributing role of asparagine in promoting cancer cell proliferation, we conducted metabolomic analysis and examined the levels of asparagine in the two cell lines using 2D monolayer culture. LC–MS analysis revealed a drastic depletion of asparagine in the HCT116 *ASNS*^−/−^ cells compared to HCT116 *ASNS*^+/+^ cells (Supplementary Fig. 1C). Taken together, these data indicate that the loss of HCT116 *ASNS* combined with depleted extracellular asparagine may be responsible for the reduced cell proliferation.

To investigate the effects of *ASNS* deletion in tumor progression, we used a cell-line derived cancer xenograft Rag2/IL2RG (R2G2) mouse model and performed a tumorigenicity study to examine tumor growth in vivo. The molecular genetics of R2G2 mice have been previously described [28–30]. To assess the tumorigenic effects of the CRISPR-edited *ASNS* clones, we injected 10 million cells each of HCT116 *ASNS*^−/−^ and the isogenic HCT116 *ASNS*^+/+^ lines to the R2G2 mice flank of 5 female mice/genotype (Fig. 1D). The growth kinetic curve revealed that the tumors formed in the HCT116 *ASNS*^−/−^ group grew slowly compared to the HCT116 *ASNS*^+/+^ mice (Fig. 1E). Tumor size differed significantly between the two groups of mice (Fig. 1F) showing a 1.47-fold, (p < 0.05) reduction in the HCT116 female *ASNS*^−/−^ animals (Fig. 1G). A gastrointestinal pathologist conducted histopathological evaluation of representative sections. 5 µm slide sections obtained from the female HCT116 *ASNS*^+/+^ and HCT116 *ASNS*^−/−^ tumor-bearing mice and confirmed that 100% of the tissue slides were derived from tumors indicating a complete tumor penetrance in both HCT116 *ASNS*^+/+^ and HCT116 *ASNS*^−/−^ mouse xenograft; tumors were poorly differentiated carcinoma, mimicking tumor growth characteristics in humans (Fig. 1H). Interestingly, qualitative assessment of the tumors showed there was desmoplasia (defined as a band of fibrous tissues intermixed within the tumor sheet) [31] observed within the tumor stroma in the HCT116 *ASNS*^+/+^ group which may be accounted for by a host response to the expanding viable tumor mass. Under high magnification (400x), we observed multiple mitotic figures within the HCT116 *ASNS*^+/+^ tumors (Fig. 1H).

Using immunohistochemistry, we observed reduced staining for Ki67, an established marker of proliferation, in the HCT116 *ASNS*^−/−^ female group to the HCT116 *ASNS*^+/+^ female group (Fig. 1I). Quantification of the mitotic figures under high-power field revealed a 75% significant (*p* < 0.01) reduction in the mitotic activity index (MAI) (Fig. 1J) and a 46.2% significant reduction (*p* < 0.0001) in Ki67 positivity in the HCT116 *ASNS*^−/−^ group (Fig. 1K). Notably, sections that were observed to be undergoing necrosis were Ki67-negative.

### *ASNS* deletion extends survival and triggers metabolic reprogramming in female R2G2 mice alone

**Fig. 2:**
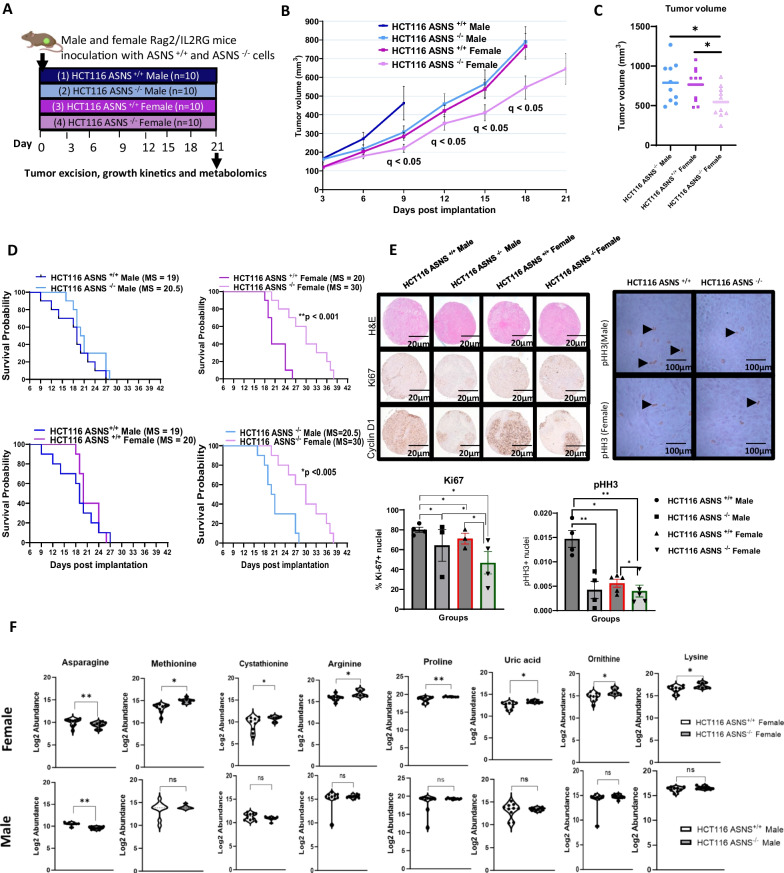
Loss of *ASNS* extends tumor-specific survival and triggers metabolic reprogramming in male and female R2G2 mice (Study 2). **A** Study design (*n* = 10 mice per group): R2G2 mice were inoculated with *HCT116* *ASNS*^+/+^ and HCT116 *ASNS*^−/−^ cells lines. Day 0 represents the day when inoculation was performed. In Group 1, male R2G2 mice received 10 million *ASNS*^+/+^ cells injected subcutaneously (s.c). Group 2 represents male mice that were implanted with 10 million HCT116 *ASNS* homozygous knockout cells. In Group 3, female R2G2 mice were implanted with HCT116 *ASNS*^+/+^ cells. Group 4 female R2G2 mice were implanted with HCT116 *ASNS*^−/−^ cells lines. **B** Tumor kinetics after cell lines implantation. Tumorigenicity experiment showing a sustained growth kinetics in the four groups after subcutaneous implantation of cell lines. **C** Tumor volume at 18 days post-tumor implantation. **D** Kaplan Meier curve indicates that loss of *ASNS* dramatically extends survival of female R2G2 mice by 11 days when compared to the ASNS^+/+^ male. **E** Representative images of colorectal cancer tissue microarray (TMA) from ASNS tumors showing immunohistochemical staining of Ki67, cyclin D1 and pHH3. Hematoxylin and eosin stained-sections are also included. *100*× magnification (Ki67, Cyclin D1 and H&E) and *400*× magnification (phospho-histone H3, pHH3). Quantitation of Ki67 and pHH3 staining in 4 groups (n = 5 mice per group). **F** Untargeted metabolomics revealed sex differences in transsulfuration and urea cycle metabolism in tumor metabolome. Box and whiskers plots display median value (center line) and whiskers showing the minimum and maximum value. Violin plots of transformed metabolite levels were generated using Graphpad prism. HCT116 *ASNS* female^+/+^ n = 10, HCT116 *ASNS*^−/−^ female n = 8, HCT116 *ASNS*^+/+^ Male n = 10, HCT116 *ASNS*^−/−^ male n = 10. Statistical significance difference was determined using two-way ANOVA with mixed-effects model for the growth kinetics in **B**, and mean tumor volumes (day 18) in **C**. Two-way ANOVA with mixed-effects model was used to accommodate the maximum tumor volume reached in several of the HCT116 *ASNS*^+/+^ male mice starting day 9 post tumor implantation. Chi-square Fisher’s exact test was used to estimate the difference in proportion of positively stained cells (**E**-**F**). Data in the bar plot are presented as mean ± SEM. For tumor growth kinetics and metabolomics data, p value was adjusted for multiple comparisons using FDR (Benjamini Hochberg) correction method. * denotes q < 0.05, and ** denotes q < 0.01

To examine whether *ASNS* specifically has any effect on survival by sex, we performed a survival study, wherein male and female R2G2 mice were inoculated subcutaneously (s.c) with 10 million cells HCT116 *ASNS*^+/+^ or HCT116 *ASNS*^−/−^ (n = 10 mice/genotype/sex) (Fig. 2A). A tumor kinetics curve revealed rapid tumor formation and growth in the HCT116 *ASNS*^+/+^ male, HCT116 *ASNS*^−/−^ male, and HCT116 *ASNS*^+/+^ female groups (Fig. 2B). In contrary, tumor growth was slower in the HCT116 *ASNS*^−/−^ female group (Fig. 2B). At day 9 post inoculation, *ASNS* depletion in the tumor xenografts from female mice was associated with a reduction (1.4-fold, q < 0.05) in tumor burden compared to the HCT116 *ASNS*^−/−^ male group (Fig. 2B) suggesting a distinct tumor phenotype in the HCT116 *ASNS* female mice during the early stages of tumor development. As the average tumor volume is reported for each group of mice, and some of the male mice with HCT116 *ASNS*^+/+^ cells had maximal tumor burden at day 9, successive measurements of tumor volume were not taken for this group. At day 18 post-inoculation, HCT116 *ASNS*^−/−^ female mice maintained a sustained reduction in tumor burden compared to the HCT116 *ASNS*^−/−^ male mice (1.45-fold, q < 0.05) (Fig. 2C). Similar to our initial xenograft in female mice only (tumorigenicity study 1), tumor volume was significantly different between the HCT116 *ASNS*^−/−^ female and HCT116 *ASNS*^+/+^ female mice (1.4-fold, q < 0.05) at 18 days post-implantation (Fig. 2C). In general, these data indicate a sex-specific difference in tumor growth that is dependent on *ASNS* activity during cancer development.

Next, we investigated whether tumor-specific survival differences exist between the two genotypes and sexes. Survival analysis revealed that there was no significant difference in the median survival (MS) between the HCT116 *ASNS*^+/+^ male and HCT116 *ASNS*^−/−^ male group (HCT116 *ASNS*^+/+^ male: MS = 19 days; HCT116 *ASNS*^−/−^ male: MS = 20.5 days, *p* = 0.22) (Fig. 2D). There was also no difference in survival between male and female mice that harbored HCT116 *ASNS*^+/+^ tumors. However, the loss of *ASNS* extended survival in female HCT116 *ASNS*^−/−^ tumor bearing mice compared to the HCT116 *ASNS*^+/+^ female group (HCT116 *ASNS*^+/+^ female: MS = 20 days; HCT116 *ASNS*^−/−^ female: MS = 30 days, *p* < 0.001) (Fig. 2D). In addition, females with HCT116 *ASNS*^−/−^ tumors had significantly improved survival compared to the male HCT116 *ASNS*^−/−^ counterpart (HCT116 *ASNS*^−/−^ male: MS = 20.5 days, HCT116 *ASNS*^−/−^ female: MS = 30 days, *p* < 0.01) (Fig. 2D). Together, these findings indicate that *ASNS* deletion induces a robust antitumor activity and extended survival in tumor-bearing female R2G2 mice; an outcome that was absent in tumor-bearing male mice.

To further understand the cellular changes associated with *ASNS* loss, we used high-throughput tissue microarrays (TMAs). Histopathological assessment of TMA showed that all tumors from male and female mice were characterized by invasive carcinoma growing in solid sheets, with no evidence of glandular differentiation, morphologically classified as high grade poorly differentiated carcinoma. We observed that male HCT116 *ASNS*^+/+^ tumors were predominantly viable, with rare foci of necrosis (Fig. 2E). Tumors from female HCT116 *ASNS*^−/−^ mice showed a sparse distribution of macrophage infiltration around the tumors. To investigate the mechanism of tumor suppression observed in the female mice with HCT116 *ASNS*^−/−^ cells, we assessed the rate of proliferation in both male and female tumors. Immunohistochemical (IHC) staining analyses revealed that female HCT116 *ASNS*^−/−^ tumors contained a significantly lower proportion of Ki67-positive cells compared to HCT116 *ASNS*^+/+^ tumors (Fig. 2E). Representative images of hematoxylin and eosin-stained TMA slides revealed a significantly reduced proportion of mitotic figures in the female HCT116 *ASNS*^−/−^ group. IHC analysis of phospho-histone H3 (pHH3), a marker of mitosis, showed a reduced number of pHH3-positive cells in the female *ASNS*^−/−^ tumor slides, confirming a lower number of mitotic figures in the HCT116 *ASNS*^−/−^ female group (Fig. 2E)*.* Quantitation of IHC stains showed a significant reduced proportion of Ki67-positive cells ( − 33.3%, *p* < 0.05) in the female HCT116 *ASNS*^−/−^ tumor bearing mice (Fig. 2E). HCT116 *ASNS*^−/−^ female tumors showed a significantly reduced number of Ki67-positive staining compared to the other three groups (HCT116 *ASNS*^−/−^ female vs. HCT116 *ASNS*^+/+^ female: − 24.4%, *p* < 0.05, HCT116 *ASNS*^−/−^ female vs HCT116 *ASNS*^+/+^male: -33.3%, *p* < 0.05), HCT116 *ASNS*^−/−^ female vs HCT116 *ASNS*^−/−^ male: − 31%, *p* < 0.05). Similarly, quantitation of pHH3 staining revealed a significantly reduced level of pHH3- marked nuclei (72.8% reduction, *p* < 0.05) within the tumors of the HCT116 *ASNS*^−/−^ female mice compared to the HCT116 *ASNS*^+/+^ male control. Notably, HCT116 *ASNS*^+/+^ tumors from female mice showed a drastic reduced number (61.9% reduction, *p* < 0.05) of mitotic figures indicated by pHH3 positivity compared to the HCT116 *ASNS*^+/+^ tumors from males (Fig. 2E), suggesting that reduced proliferation and mitosis are associated with the observed tumor suppression in the tumor-bearing female mice with HCT116 *ASNS*^−/−^ xenograft. To validate macrophage infiltration, we performed immunostaining for F4/80, a macrophage selective marker. F4/80^+^ cells were observed within viable tumors of all xenografts from male and female mice (n = 5 samples/group) (Supplementary Fig. 2). We observed a higher level of F4/80 staining in both HCT116 *ASNS*^−/−^ groups compared to the control HCT116 *ASNS*^+/+^ groups (Supplementary Fig. 2).

### ASNS deletion alters tumor metabolism in a sex-specific manner

Since we hypothesized that the link between *ASNS* and poorer survival in female CRC patients is due to sex-specific metabolic reprogramming, we performed metabolomics analysis on male and female tumor samples taken from R2G2 mice in the survival study (n = 10/group). A full list of differentially altered metabolites between HCT116 *ASNS*^*−/−*^ and HCT116 *ASNS*^+*/*+^ tumors is presented with respect to sex of the mouse in Supplementary Table S1. There were no commonly dysregulated metabolites between the male and female tumor bearing R2G2 mice (q < 0.05). Interestingly, there were 35 significantly altered metabolites (identified in HILIC mode) in the female R2G2 mice when comparing HCT116 *ASNS*^−/−^ to HCT116 *ASNS*^+/+^ tumors. In comparison, one metabolite was identified in males that was significantly altered in RPLC mode (q < 0.05) (Supplementary Table S1).

The metabolites that were altered in female tumors broadly represented metabolic changes to the transsulfuration pathway, urea cycle and gluconeogenesis. Deletion of *ASNS* caused a reduction in tumor asparagine levels and there was no sex difference in asparagine levels after the gene deletion (Fig. 2F). However, HCT116 *ASNS*^−/−^ female tumors had a significantly higher abundance of methionine (q = 0.007), cystathionine (q = 0.09) and lysine (q = 0.076) compared to HCT116 *ASNS*^+/+^ female tumors, suggesting an upregulation of the transsulfuration pathway to maintain redox balance (Fig. 2F, Supplementary Table S1). Three metabolites involved in the urea cycle also had increased abundance in HCT116 *ASNS*^−/−^ tumors: uric acid (q = 0.04), ornithine (q = 0.049), and arginine (q = 0.039). Arginine can serve as a substrate for proline synthesis [32], and proline was also observed to be increased in the HCT116 *ASNS*^−/−^ female tumors (q = 0.047). (Fig. 2F, Supplementary Table S1). Further investigation of tumor amino acid levels showed that other amino acids were significantly increased in HCT116 *ASNS*^−/−^ tumors from females only: serine (q = 0.07) alanine (q = 0.04), phenylalanine (q = 0.039) and methionine sulfoxide (MetO), an oxidized form of methionine (q = 0.031) (Supplementary Fig. 3, Supplementary Table S1). In addition, a number of acetylated amino acids, and di- and tripeptides, were increased (Supplementary Fig. 3, Supplementary Table S1). Increased levels of lactate were observed in HCT116 *ASNS*^−/−^ tumors from female mice (compared to HCT116 *ASNS*^+/+^) (q = 0.012) which in addition to alanine are metabolites that can serve as a gluconeogenic precursors to phosphoenolpyruvate. Levels of phosphoenolpyruvate were also significantly increased in the HCT116 *ASNS*^−/−^ female tumors compared to the HCT116 *ASNS*^+/+^ female tumors (q = 0.028) (Supplementary Fig. 3). In males, only leukotriene B4 (LTB4) was increased (q < 0.05) in tumors from HCT116 *ASNS*^−/−^ xenograft, and this metabolite was not altered in female tumor xenografts (in Supplementary Fig. 2). Using Ingenuity pathway analysis (IPA), we performed a network analysis to determine the relationships between the altered metabolites and possible interactions with signaling molecules. We observed that a number of the alterations observed in amino acid levels were linked to upregulation of amino acid transporters (SLC38A3, SLC6A14, SLC25A29, SLC7A5) and G-protein coupled receptor isoform 6A (GPRC6A) (Supplementary Fig. 4). A predicted inhibition of PI3K and c-Jun N-terminal kinase (Jnk) was observed (JNK sits upstream of *ASNS* regulation), and also the activation of ribosomal protein S6 kinase beta-1 (p70S6k), protein kinase B (Akt), p38 mitogen-activated protein kinase (P38 MAPK), and extracellular signal-regulated kinase (ERK) (Supplementary Fig. 4). It was not possible to perform a network analysis using the data from male mice due to the low number of significantly altered metabolites. Overall, these findings indicate that tumors from female mice have complex metabolic rewiring as a consequence of *ASNS* knockout, which is not seen in tumors from male mice, showing that *ASNS* may have sex-specific actions on CRC metabolism.

### Transcriptomic profiling of ***ASNS***^+/+^ and ***ASNS***^−/−^ tumors identifies ***ASNS*** and SLC1A3 as tumor enhancers

**Fig. 3:**
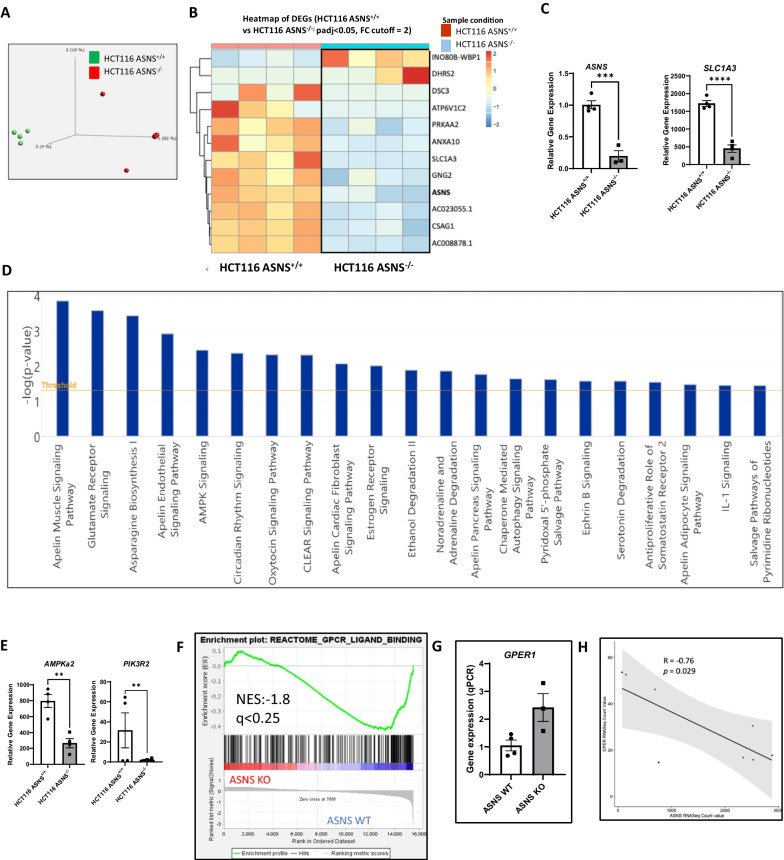
Transcriptomic profiling of HCT116 cell-line derived tumors identifies genes related to *ASNS* in females. **A** Principal component analysis (PCA) shows a unique separation of *ASNS*^−/−^ samples from the *ASNS*^+/+^ control using normalized count (n = 4 mice/genotype). **B** Heatmap reveals the top 12 differentially expressed genes using a fold change cut off = 2 after adjusting for multiple comparisons. **C** Validation of gene expression using RT-qPCR for asparagine synthetase (*ASNS*), RNA sequencing revealed the downregulation of the glutamate aspartate transporter 1 (*SLC1A3*). **D** Ingenuity pathway analysis (IPA) revealed metabolic pathways impacted by *ASNS* signaling disruption. **E**. Analysis of top differentially expressed genes revealed that the key metabolic genes Protein Kinase AMP-Activated Catalytic Subunit Alpha 2 (*AMPKa2*) and Phosphoinositide-3-Kinase Regulatory Subunit 2 (*PIK3R2*) were significantly (p < 0.05) downregulated in the female ASNS^−/−^ tumors. **F** Gene set enrichment analysis (GSEA) revealed GPCR ligand binding enriched in the HCT116 *ASNS*^+/+.^with a normalized enrichment score (NES) of − 1.8 and a q value of 0.16 **G.** Relative mRNA expression of *GPER1* in the HCT116 *ASNS*^+/+^ female and HCT116 *ASNS*^−/−^ female tumors. **H.** Correlation between *ASNS* and *GPER1* using RNA Seq count value. PCA was conducted using normalized log count of annotated genes to generate plot on Qlucore v3.8. Normalization was set to Mean = 0 and variance = 1. q value was set as 0.1. Heatmap showing DEGs from DESEQ2 on R studio. (HCT116 *ASNS*^+/+^, n = 4 and HCT116 *ASNS*^−/−^, n = 4). Individual dot plot represents mean ± SEM with *p* value < 0.05 considered to be significant using two-sided *t*-test. * represents *p* < 0.05 and **denotes *p* < 0.01. An outlier in the *ASNS*^−/−^ group was excluded from the *TNFRSF9* dot plot due to the non-detectability of *TNFRSF9* in 75% of tumor samples in the *ASNS*^−/−^ group. For all qPCR experiments, an outlier in HCT116 *ASNS*^−/−^ group was also excluded due to the non-detectability of RNA transcripts in one sample (CT value > 36). Canonical pathways were identified using a –log (*p*-value) score cutoff of 1.4 and Fisher’s Exact Test on the IPA program. Linear regression plot was generated using R studio. GSEA was conducted using normalized count data and an FDR q value cut off < 25%

Since we identified a distinct metabolic rewiring in the tumors from female R2G2 mice, and only the female tumor bearing mice with HCT116 *ASNS*^−/−^ had improved survival, we then aimed to understand the transcriptional effect of *ASNS* deletion in the female tumor bearing mice. We performed RNA-Sequencing of the HCT116 *ASNS*^+/+^ and HCT116 *ASNS*^−/−^ tumors derived from the female mice (n = 4/genotype) in the tumorigenicity study. PCA scores plots showed a distinct clustering of samples in the control HCT116 *ASNS*^+/+^ group, indicating analytical reproducibility (Fig. 3A). We identified 16 genes (12 downregulated genes and 4 upregulated genes) that were differentially expressed with a q value < 0.1 (Supplementary Table S2). Of these, 12 genes fell under our cutoff threshold of q < 0.05 and log FC ≥ 2 (Fig. 3B). As predicted, *ASNS* was identified as a DEG that was significantly enriched in the HCT116 *ASNS*^+/+^ tumors. It is noteworthy that glutamate aspartate transporter 1 (*SLC1A3*), the gene product responsible for aspartate/glutamate uptake, was also decreased. *SLC1A3* was significantly downregulated in the HCT116 *ASNS*^−/−^ cells compared to the control, suggesting reduced aspartate transport in the HCT116 *ASNS*^−/−^ tumors. Several key genes regulating metabolism (including *ASNS, SLC1A3, DSC3, ATP6V1C2*)*,* transcriptional events and oncogenic signaling *(PRKAA2 (*also known as AMPK*), ANXA10, GNG2*, and *CSAG1)*) were significantly downregulated by *ASNS* deletion in the female CRC (Fig. 3B). To verify the data from RNA-Seq, we conducted rt-qPCR to quantify the expression levels of *ASNS* and *SLC1A3* which was downregulated in the HCT116 *ASNS*^−/−^ tumors compared to HCT116 *ASNS*^+/+^ group (Fig. 3C).

Metabolic pathways and networks impacted by the loss of *ASNS* in the female tumors were further investigated using IPA, an orthogonal software. Twenty-one canonical pathways were identified as impacted in the *ASNS* CRISPR-edited tumor clones. Asparagine biosynthesis and AMPK signaling were significantly impacted in the HCT116 *ASNS*^−/−^ female group (Fig. 3D), indicating that the loss of *ASNS* alters the metabolic profile and oncogenic signaling of female tumors.

Surprisingly, *ASNS* deletion caused a dramatic reduction in *PIK3R2* which was predicted to be inhibited in the metabolomics analysis. In addition, RNA-seq analysis showed a decrease of *PRKAA2 (*also known as *AMPK)* that was predicted to be linked to PIK3CA (Fig. 3E). To further explore the impacts of these potential markers of cancer, network analysis revealed the interconnectivity of these genes. Interestingly, ERK was predicted to be activated which validates the findings from the metabolomics network analyses (Supplementary Figs. 4 and 5).

In addition to network analysis, we also performed gene set enrichment analysis (GSEA) which revealed that G-protein coupled receptor (GPCR) ligand binding was one of the top 50 enriched gene set with a normalized enrichment score of − 1.8 (q = 0.16, *p* < 0.05) in the HCT116 *ASNS*^+*/*+^ female tumors. Specifically, G protein-coupled estrogen receptor 1 (also known as *GPER1*) was significantly upregulated in the *ASNS*^−/−^ tumors (Fig. 3F, 3G). Of note, estrogen receptor signaling was one of the canonical pathways altered in Fig. 3D. Validation of *GPER1* expression was performed using rt-qPCR and we observed a striking 2.4-fold (*p* = 0.035) upregulation of *GPER1* mRNA in the HCT116 *ASNS*^−/−^ tumors compared to HCT116 *ASNS*^+/+^ tumors from female mice (Fig. 3G). Regression analysis confirmed a strong negative correlation between *GPER1* and *ASNS* expression in female HCT116 *ASNS*^−/−^ tumors (r = − 0.76, *p* = 0.029) (Fig. 3H), thus suggesting a possible link between *GPER1* activation and *ASNS* signaling. We also examined the nuclear estrogen receptors *ESR1* and *ESR2*. *ESR1* which codes for Erα was not expressed, which was expected as Erα expression is lost during colon tumorigenesis [33], and there was no difference in relative *ESR2* expression (data not shown). Together, these data indicate that *ASNS* deletion exhibited pronounced effects on important metabolic pathways and hormone receptor signaling in tumor-xenografts implanted into female R2G2 mice. Validation of the transcriptomic changes in the larger mice cohort which included both male and female xenograft mice (n = 8–10 mice per group) showed that *ASNS* was significantly downregulated in the HCT116 *ASNS*^−/−^ tumor xenografts from male and female R2G2 mice (Supplementary Figs. 6). In contrast, *GPER1* was upregulated in the HCT116 *ASNS*^−/−^ compared to HCT116 *ASNS*^+/+^ tumor xenografts in both male and female mice (Supplementary Figs. 6).

### Disruption of *ASNS* signaling synergizes with the novel G-protein coupled estrogen receptor to inhibit tumor progression

**Fig. 4:**
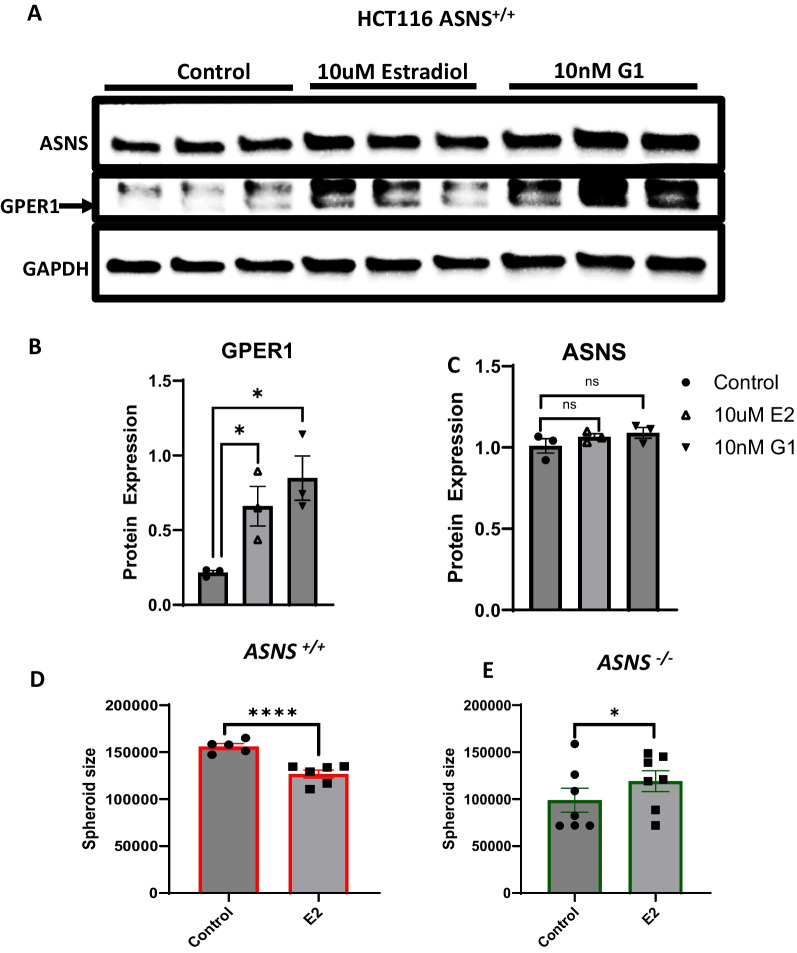
Relationship between asparagine synthetase (ASNS) and G-protein coupled estrogen receptor (GPER) signaling. **A** Western blot indicating ASNS and GPER1 expression in estradiol (E2)-treated *ASNS*^+/+^ cell lines. RPMI 1640 containing 0.4 mM of asparagine and supplemented with 4mM glutamine was used for in vitro experiments. G1, a GPER agonist, serves as a positive control for the western blot experiment. **B** Quantitation of GPER1 levels in E2-treated *ASNS*^+/+^ cell line. **C** Quantitation of ASNS levels in in E2- treated *ASNS*^+/+^ cell line *n* = 3 replicates per condition **D** Estradiol treatment of HCT116 *ASNS*^+/+^ cells under normal nutrient condition. Cells were treated with media supplemented E2 (10 µM). **E** Estradiol treatment of HCT116 *ASNS*^−/−^ cells under sufficient nutrient condition. Individual dot plots represent mean ± SEM. Statistical significance was determined using student *t*-test with *indicating *p* < 0.05, **indicating *p* < 0.01, *** indicating *p* < 0.001 and **** indicating *p* < 0.0001 after multiple comparison (Benjamini Hochberg’s FDR method). ANOVA was used to compare differences in mean values of the E2, G1 and control groups (**B** and **C**). Quantification of band intensity was conducted using Image J software

As GPCR ligand binding was enriched in the comparison of HCT116 *ASNS*^*−/−*^ and HCT116 *ASNS*^+*/*+^ tumors, and *GPER1* negatively correlates with *ASNS* expression, we sort to understand the effect of estradiol, a ligand for *GPER1*, on both *GPER1* and *ASNS* expression. Estradiol is a sex-steroid hormone that is produced in both males and females, it is known to exert anti-proliferative effects in CRC, and is produced at 10-times higher levels in females premenopause compared to age-matched men GPER1 is a membrane bound protein that mediates the biological effects of estrogen. Therefore, examination of the effect of E2 on GPER1 and ASNS could help understand the link between sex sex-related factors that may affect ASNS-expressing tumor cells and attenuated tumor growth in females. E2 has been shown to reduce cancer cell migration and estrogenic signaling, an effect that is dependent on GPER1 activity; of note, these changes are reversed by *GPER1* silencing under hypoxic conditions [16, 34]. We examined whether E2 can activate *GPER1* in HCT116 *ASNS*^+/+^ cells (that have low GPER1 expression). As a positive control we used G1, a selective *GPER1* agonist. Analysis of protein lysates from both E2- and G1-treated HCT116 *ASNS*^+*/*+^ cells using western blot confirmed GPER1 activation. (Fig. 4A-B). 10 μM E2 increases GPER1 expression in the HCT116 *ASNS*^+*/*+^ cells compared to untreated control. The levels of ASNS were not different between E2-treated cells, or control groups (Fig. 4A-C). HCT116 *ASNS*^+/+^ and HCT116 ASNS^-/-^ cells were further cultured in E2 and spheroid size measured. HCT116 *ASNS*^+/+^ culture with E2, which increased GPER1, showed a decrease in cell growth, aligning with previous results that show HCT116 ASNS^−/−^ tumors with high GPER1 have decreased tumor size. However when HCT116 *ASNS*^*−*/−^ cells were cultured in E2, an increase in tumor growth was observed (Figs. 4B-C). Overall, these results indicate that the anti-proliferative effects of E2 which may be mediated through ligand-activation of GPER1, are dependent on ASNS expression.

## Discussion

We have previously reported sex-specific associations between ASNS, asparagine levels and patient survival [14, 35]. The mechanisms underlying these findings are not clearly understood. Asparagine plays several biological roles such as maintaining cellular homeostasis, and it also fuels other amino acid metabolic pathways [15, 36]. Given these previous findings, we examined the role of *ASNS* in tumorigenesis and its effects on tumor metabolism, using a cell line derived xenograft model of CRC. Colon cancer cells were implanted into the flank of male and female R2G2 mice. We observed a higher tumor burden in HCT116 *ASNS*^+/+^ tumor-bearing male mice and shortened survival specifically in HCT116 *ASNS*^+/+^ and HCT116 *ASNS*^−/−^ tumor-bearing male relative to female mice. Furthermore, we used a combined approach of histology, IHC, transcriptomics, and metabolomics to gain insights into how *ASNS*-driven asparagine metabolism may regulate colon carcinogenesis.

Examination of  tissue slides from the male HCT116 *ASNS*^+/+^ tumors displayed a higher abundance of fibrotic connective tissues and multiple mitotic figures. It has been previously reported that fibrotic connective tissues are associated with tumor invasiveness and are a potential prognostic marker of CRC metastasis [37, 38]. In contradistinction, HCT116 *ASNS*^*−/−*^ tumors from female mice displayed reduced pHH3 staining and low Ki67 positivity confirming that the protection observed in the female mice is due to reduced proliferation, wherein *ASNS* loss exerts a dampening effect on the cell cycle. In addition, tumors from female HCT116 *ASNS*^*−/−*^ tumors showed a higher degree of infiltrating leucocytes and cancer associated fibroblasts with scarce viable tumors compared to all tumors from male mice. An abundant distribution of intraepithelial lymphocytes is often correlated with an onset of necrosis [39]. Notably, tumors from female HCT116 *ASNS*^−/−^ mice showed significant necrosis compared to the other groups of mice.

Transcriptomic analysis also revealed other notable changes at the gene expression level by *ASNS* genotype. *SLC1A3* was significantly reduced in the female HCT116 *ASNS*^*−/−*^ tumors compared to HCT116 *ASNS*^+*/*+^ tumors. *SLC1A3* is known to contribute towards tumor development in human patients with metastatic CRC [40]. *SLC1A3* also allows cancer cells to take up amino acids and maintains a bidirectional interaction with the tumor microenvironment which can promote hyperproliferation and tumor progression [39]. Pharmacologic blockade of SLC1A3 sensitizes cancer cells to electron transport chain inhibitors and induces cell death [41]. Given that *SLC1A3* is reduced in HCT116 *ASNS*^−/−^ tumors, this favorably predisposes cancer cells, with reduced amino acid uptake to cell death by necrosis. In addition to SLC1A3, another cotransporter, SLC5A1 was also downregulated in HCT116 *ASNS*^−/−^ tumors. *SLC5A1*, which is expressed in the intestines, encodes the sodium-glucose cotransporter (SGLT1), and carries glucose into cells against a sodium ion concentration gradient [42]. Given that this gene is downregulated, it supports findings from the metabolomics analysis that these tumors have altered glucose metabolism and favor gluconeogenesis.

Untargeted metabolomics analysis revealed that metabolites involved the transsulfuration pathway (methionine**,** cystathionine, and lysine which support cell death), the urea cycle (uric acid, ornithine, arginine, and proline), and gluconeogenesis (lactate, alanine and phosphoenolpyruvate), were all increased in female tumors with HCT116 *ASNS*^−/−^ compared to HCT116 *ASNS*^+/+^ cells. There was no alteration in these metabolites between the two *ASNS* genotypes from male tumors. This was a particularly interesting finding given that the HCT116 cell line is derived from a male patient, therefore the metabolomic changes observed in the tumors results from factors extrinsic to the tumor that are related to the sex of the mouse. An unexpected finding was an increase in gluconeogenesis in the HCT116 *ASNS*^−/−^ tumors. Increased gluconeogenesis has been shown to inhibit glycolysis and tumor growth [43]. Given that our pathway analyses of both metabolite and transcriptomics data showed a reduction of *AMPK*, an important mediator of nutrient supply, it is evident that *ASNS* knockout in female tumors has a marked decrease in nutrient supply that may be affecting tumor growth. These findings also support our prior study which hypothesized that CRCs from female patients have a nutrient deplete phenotype [14]. The increase in both gluconeogenesis and the urea cycle also suggest increased disposal of amino acids that are no longer needed physiologically by the tumor for growth, wherein excess amino acids are converted into urea through the urea cycle, or transformed into other intermediates such as glucose when the supply of glucose is insufficient during metabolic stress or starvation [44]. Overall, the sex-specific differences in metabolite levels caused by ASNS, suggests a mechanism by which loss of *ASNS* can increase critical metabolites to confer protection and improve survival outcomes in female mice, possibly by decreasing the expression of important nutrient sensors. To our knowledge, this is the first study to show that *ASNS* deletion is particularly important in altering widespread metabolism in females only and not males, and is due to tumor-extrinsic factors. This distinct “metabotype” can explain the sex-specific difference in colon tumorigenesis.

Within our study we  showed that *GPER1* expression increased in HCT116 *ASNS*^−/−^ tumors. *GPER1* is intricately linked to nutrient status and hormone levels, furthermore it has been poorly characterized with respect to CRC progression [45]. As our study examined sex-differences we further examined whether *GPER1* is activated by estradiol in vitro. Interestingly, treatment with E2 caused an increase in *GPER1* in HCT116 *ASNS*^+/+^ cells, and a decrease in tumor spheroid size was observed. However treatment of E2 with HCT116 *ASNS*^−/−^ cells caused an increase in spheroid size. These results indicate that there is an complex interaction between E2, ASNS and GPER1 that required further mechanistic investigation. It is however possible that *GPER1* and *ASNS* are linked through KRAS; *GPER1* regulates phosphoinositide-3-kinase (PI3K) which sits downstream of *KRAS* [16].

Indeed, the functional roles of *GPER1* are not entirely understood especially within the context of therapeutic design [46]. Conflicting reports exist for the role estrogen-mediated *GPER1* activation in cancer progression plays. One study showed that E2 can act through *GPER1* to enhance hypoxia-induced expression of *HIF1-α* and *VEGF* expression to promote CRC cell migration and proliferation [34]. However, it has also been shown that treatment of a mouse model of ulcerative colitis (UC) with G1 resolves UC and protects colon crypt cells [47].

Finally, we acknowledge that a limitation of this study is that the subcutaneous xenograft model does not completely recapitulate human CRC growing within the colorectum [48]. The absence of T cells in the immunocompromised R2G2 mice may also limit the translational applicability of our model [48, 49].

## Conclusions

The omics approach utilized in this study revealed that sex-specific variability exists for CRC. Additionally, there is an implication for sex in relationship to the transcriptome and metabolome of whole organisms under physiological and pathological conditions. Future studies will aim to evaluate the relevance of dietary asparagine depletion and supplementation on CRC progression. The pronounced effect of *ASNS* deletion on suppressing tumor size and volume is promising for the potential targeting of *ASNS*, and the potential role of E2 and *GPER1* mediating this effect is an area of future investigation. Overall, our data demonstrate that multivariate approach that integrates metabolomic profiling, transcriptomics and immunochemical analysis can capture the dynamics of pathological states, which are essential for explaining the sex-based differences in CRC.

### Supplementary Information

Supplementary Material 1.

Supplementary Material 2.

## Data Availability

The authors declare that all relevant data used to conduct the study are within this article. RNA-Sequencing data are stored on the Yale High Performance Computing cluster and will be made available upon request.

## Code availability

Code used for the untargeted metabolomics analysis will be made available upon request.

## References

[1] Siegel RL, Miller KD, Goding Sauer A, Fedewa SA, Butterly LF, Anderson JC (2020). Colorectal cancer statistics, 2020. CA Cancer J Clin.

[2] Siegel RL, Miller KD, Fuchs HE, Jemal A (2021). Cancer Statistics, 2021. CA Cancer J Clin.

[3] Signorelli C, Chilelli MG, Sperduti I, Giacinti S, Amodio PM, Palmieri RM (2019). Correlation of tumor location to clinical outcomes in colorectal cancer: a single-institution retrospective analysis. Anticancer Res.

[4] Cai Y, Shen X, Lu L, Yan H, Huang H, Gaule P (2022). Bile acid distributions, sex-specificity, and prognosis in colorectal cancer. Biol Sex Differ.

[5] Breivik J, Meling GI, Spurkland A, Rognum TO, Gaudernack G (1994). K-ras mutation in colorectal cancer: relations to patient age, sex and tumour location. Br J Cancer.

[6] Nelson HH, Christiani DC, Mark EJ, Wiencke JK, Wain JC, Kelsey KT (1999). Implications and prognostic value of K-ras mutation for early-stage lung cancer in women. J Natl Cancer Inst.

[7] Shin JY, Jung HJ, Moon A (2019). Molecular markers in sex differences in cancer. Toxicol Res.

[8] Charitou T, Srihari S, Lynn MA, Jarboui MA, Fasterius E, Moldovan M (2019). Transcriptional and metabolic rewiring of colorectal cancer cells expressing the oncogenic KRAS(G13D) mutation. Br J Cancer.

[9] Toda K, Kawada K, Iwamoto M, Inamoto S, Sasazuki T, Shirasawa S (2016). Metabolic alterations caused by KRAS mutations in colorectal cancer contribute to cell adaptation to glutamine depletion by upregulation of asparagine synthetase. Neoplasia.

[10] Pavlova NN, Hui S, Ghergurovich JM, Fan J, Intlekofer AM, White RM (2018). As extracellular glutamine levels decline, asparagine becomes an essential amino acid. Cell Metab..

[11] Kawada K, Toda K, Sakai Y (2017). Targeting metabolic reprogramming in KRAS-driven cancers. Int J Clin Oncol.

[12] Kan CC, Chung TY, Juo YA, Hsieh MH (2015). Glutamine rapidly induces the expression of key transcription factor genes involved in nitrogen and stress responses in rice roots. BMC Genomics.

[13] Lopes-Ramos CM, Quackenbush J, DeMeo DL (2020). Genome-wide sex and gender differences in cancer. Front Oncol.

[14] Cai Y, Rattray NJW, Zhang Q, Mironova V, Santos-Neto A, Hsu KS (2020). Sex differences in colon cancer metabolism reveal a novel subphenotype. Sci Rep.

[15] Shen X, Jain A, Aladelokun O, Yan H, Gilbride A, Ferrucci LM (2022). Asparagine, colorectal cancer, and the role of sex, genes, microbes, and diet: a narrative review. Front Mol Biosci.

[16] Lu L, Zhang Q, Shen X, Zhen P, Marin A, Milian RG, et al. Asparagine synthetase and G-protein coupled estrogen receptor are critical responders to nutrient supply in KRAS mutant colorectal cancer. bioRxiv. 2023. 10.1002/ijc.35104 PMC11537827 39039782

[17] Ogola BO, Abshire CM, Visniauskas B, Kiley JX, Horton AC, Clark-Patterson GL (2022). Sex differences in vascular aging and impact of GPER deletion. Am J Physiol Heart Circ Physiol.

[18] Pietras RJ, Szego CM (1977). Specific binding sites for oestrogen at the outer surfaces of isolated endometrial cells. Nature.

[19] Molina L, Figueroa CD, Bhoola KD, Ehrenfeld P (2017). GPER-1/GPR30 a novel estrogen receptor sited in the cell membrane: therapeutic coupling to breast cancer. Expert Opin Ther Targets.

[20] Baboo J, Kilbride P, Delahaye M, Milne S, Fonseca F, Blanco M (2019). The impact of varying cooling and thawing rates on the quality of cryopreserved human peripheral blood T cells. Sci Rep.

[21] Yang S, Wang X, Contino G, Liesa M, Sahin E, Ying H (2011). Pancreatic cancers require autophagy for tumor growth. Genes Dev.

[22] Staff PO (2017). Correction: a novel intraperitoneal metastatic xenograft mouse model for survival outcome assessment of esophageal adenocarcinoma. PLoS ONE.

[23] Peng X, Kim J, Gupta G, Agaronyan K, Mankowski MC, Korde A (2022). Coronavirus lung infection impairs host immunity against secondary bacterial infection by promoting lysosomal dysfunction. J Immunol.

[24] Subramanian A, Tamayo P, Mootha VK, Mukherjee S, Ebert BL, Gillette MA (2005). Gene set enrichment analysis: a knowledge-based approach for interpreting genome-wide expression profiles. Proc Natl Acad Sci USA.

[25] Langlois MJ, Bergeron S, Bernatchez G, Boudreau F, Saucier C, Perreault N (2010). The PTEN phosphatase controls intestinal epithelial cell polarity and barrier function: role in colorectal cancer progression. PLoS ONE.

[26] Wang Z, Li Y, Liu ET, Yu Q (2004). Susceptibility to cell death induced by blockade of MAPK pathway in human colorectal cancer cells carrying Ras mutations is dependent on p53 status. Biochem Biophys Res Commun.

[27] Harma V, Virtanen J, Makela R, Happonen A, Mpindi JP, Knuuttila M (2010). A comprehensive panel of three-dimensional models for studies of prostate cancer growth, invasion and drug responses. PLoS ONE.

[28] Park SH, Kyin T, Bendelac A, Carnaud C (2003). The contribution of NKT cells, NK cells, and other gamma-chain-dependent non-T non-B cells to IL-12-mediated rejection of tumors. J Immunol.

[29] Nanni P, Nicoletti G, Palladini A, Croci S, Murgo A, Ianzano ML (2012). Multiorgan metastasis of human HER-2+ breast cancer in Rag2-/-;Il2rg-/- mice and treatment with PI3K inhibitor. PLoS ONE.

[30] Eisenring M, vom Berg J, Kristiansen G, Saller E, Becher B (2010). IL-12 initiates tumor rejection via lymphoid tissue-inducer cells bearing the natural cytotoxicity receptor NKp46. Nat Immunol.

[31] Wolf B, Weydandt L, Dornhofer N, Hiller GGR, Hohn AK, Nel I (2023). Desmoplasia in cervical cancer is associated with a more aggressive tumor phenotype. Sci Rep.

[32] Lieu EL, Nguyen T, Rhyne S, Kim J (2020). Amino acids in cancer. Exp Mol Med.

[33] Cleveland AG, Oikarinen SI, Bynote KK, Marttinen M, Rafter JJ, Gustafsson JA (2009). Disruption of estrogen receptor signaling enhances intestinal neoplasia in Apc(Min/+) mice. Carcinogenesis.

[34] Bustos V, Nolan AM, Nijhuis A, Harvey H, Parker A, Poulsom R (2017). GPER mediates differential effects of estrogen on colon cancer cell proliferation and migration under normoxic and hypoxic conditions. Oncotarget.

[35] Shen X, Cai Y, Lu L, Huang H, Yan H, Paty PB (2022). Asparagine metabolism in tumors is linked to poor survival in females with colorectal cancer: a cohort study. Metabolites.

[36] Vettore L, Westbrook RL, Tennant DA (2020). New aspects of amino acid metabolism in cancer. Br J Cancer.

[37] Cox TR, Erler JT (2014). Molecular pathways: connecting fibrosis and solid tumor metastasis. Clin Cancer Res.

[38] Thomas D, Radhakrishnan P (2019). Tumor-stromal crosstalk in pancreatic cancer and tissue fibrosis. Mol Cancer.

[39] Vayrynen SA, Vayrynen JP, Klintrup K, Makela J, Karttunen TJ, Tuomisto A (2016). Clinical impact and network of determinants of tumour necrosis in colorectal cancer. Br J Cancer.

[40] Li J, Lan Z, Liao W, Horner JW, Xu X, Liu J (2023). Histone demethylase KDM5D upregulation drives sex differences in colon cancer. Nature.

[41] Garcia-Bermudez J, Baudrier L, La K, Zhu XG, Fidelin J, Sviderskiy VO (2018). Aspartate is a limiting metabolite for cancer cell proliferation under hypoxia and in tumours. Nat Cell Biol.

[42] Harada N, Inagaki N (2012). Role of sodium-glucose transporters in glucose uptake of the intestine and kidney. J Diabetes Investig.

[43] Khan MW, Chakrabarti P (2015). Gluconeogenesis combats cancer: opening new doors in cancer biology. Cell Death Dis.

[44] Schutz Y (2011). Protein turnover, ureagenesis and gluconeogenesis. Int J Vitam Nutr Res.

[45] Lappano R, Rosano C, Santolla MF, Pupo M, De Francesco EM, De Marco P (2012). Two novel GPER agonists induce gene expression changes and growth effects in cancer cells. Curr Cancer Drug Targets.

[46] Prossnitz ER, Hathaway HJ (2015). What have we learned about GPER function in physiology and disease from knockout mice?. J Steroid Biochem Mol Biol.

[47] Wang Q, Li Z, Liu K, Liu J, Chai S, Chen G (2021). Activation of the G protein-coupled estrogen receptor prevented the development of acute colitis by protecting the crypt cell. J Pharmacol Exp Ther.

[48] Guerin MV, Finisguerra V, Van den Eynde BJ, Bercovici N, Trautmann A (2020). Preclinical murine tumor models: a structural and functional perspective. Elife.

[49] McDaniel Mims B, Grisham MB (2018). Humanizing the mouse immune system to study splanchnic organ inflammation. J Physiol.

