# Supplementary material for: Growth characteristics of HCT116 xenografts lacking asparagine synthetase vary according to sex
Source: Hum Genomics. 2024 Jun 17;18:67. doi: 10.1186/s40246-024-00635-3 (PMC11184737; doi:10.1186/s40246-024-00635-3)
Supplement: Supplementary file 1 — Supplementary Material 1. [file 40246_2024_635_MOESM1_ESM.pdf]

Supplementary Information for:

Growth characteristics of HCT116 xenografts lacking asparagine synthetase vary according to sex

Oladimeji Aladelokun[1], Lingeng Lu[2], Jie Zheng[1], Hong Yan[1], Abhishek Jain[1], Joanna Gibson[4], Sajid A. Khan*[3], Caroline H. Johnson*[1]

[1]*Department of Environmental Health Sciences, Yale School of Public Health, Yale University, New Haven, Connecticut*

[2]*Department of Chronic Disease Epidemiology, Yale School of Public Health, Yale University, New Haven, CT, 06510, USA*

[3]*Division of Surgical Oncology, Department of Surgery, Yale School of Medicine, New Haven, Connecticut*

[4]*Department of Pathology, Yale University, New Haven, Connecticut, USA.*

*authors to whom correspondence should be addressed
Caroline.johnson@yale.edu
Sajid.khan@yale.edu

Supplementary Materials and Method

RNA Sequencing

RNA Seq Quality Control: Total RNA quality for A260/A280 and A260/A230 ratios were performed by nanodrop. RNA integrity is determined by running an Agilent Bioanalyzer gel, which measures the ratio of the ribosomal peaks. RIN values of >/=7 or greater was required for library prep.

RNA Seq Library Prep: mRNA was purified from approximately 200ng of total RNA with oligo-dT beads and sheared by incubation at 94C in the presence of Mg (Roche Kapa mRNA Hyper Prep Cat# KR1352, Basel, Switzerland). Following first-strand synthesis with random primers, second strand synthesis and A-tailing was performed with dUTP for generating strand-specific sequencing libraries. Adapter ligation with 3’ dTMP overhangs were ligated to library insert fragments. Library amplification amplifies fragments carrying the appropriate adapter sequences at both ends. Strands marked with dUTP were not amplified. Indexed libraries are quantified by qRT-PCR using a commercially available kit (Roche KAPA Biosystems Cat# KK4854, Basel, Switzerland) and insert size distribution determined by the Agilent Bioanalyzer. Samples with a yield of ≥ 0.5 ng/ul and a size distribution of 150-300bp are used for sequencing.

Flow Cell Preparation and Sequencing: Sample concentrations were normalized to 1.2 nM and loaded onto an Illumina NovaSeq flow cell at a concentration that yields 25 million passing filter clusters per sample. Samples were sequenced using 100bp paired-end sequencing on an Illumina NovaSeq6000 according to Illumina protocols. The 10bp unique dual index is read during additional sequencing reads that automatically follow the completion of read 1. Data generated during sequencing runs were simultaneously transferred to the YCGA high-performance

computing cluster. A positive control (prepared bacteriophage Phi X library) provided by Illumina was spiked into every lane at a concentration of 0.3% to monitor sequencing quality in real time.

Pre-processing of RNA-Seq Data and Storage: Signal intensities were converted to individual base calls during a run using the system's Real Time Analysis (RTA) software. Base calls were transferred from the machine's dedicated personal computer to the Yale High Performance Computing cluster via a 1 Gigabit network mount for downstream analysis. Primary analysis – sample de-multiplexing and alignment to the human genome – was performed using Illumina's CASAVA 1.8.2 software suite. The error rate was less than 2% and the distribution of reads per sample in a lane was within reasonable tolerance.

RNA-Seq data processing

Low quality reads with Phred quality score (Q) < 20 were trimmed, and adaptor contamination were removed using Trim Galore (v0.5.0). The mean quality per read score was Q= 36 for all ten (10) samples, indicating an accurate base calling. The average number and standard deviation (SD) of reads and standard deviation was 68,849,632 ± 4,125,887 and 64,993,550 ± 4,479,456 for HCT116 *ASNS*$^{+/+}$ and HCT116 *ASNS*$^{-/-}$ respectively. After trimming, the mean number and SD of uniquely mapped reads was 52,955,285 ± 4,825,583 and 39,523,081 ± 19,704,450 for ASNS$^{+/+}$ and ASNS$^{-/-}$ respectively. Two samples with very low read counts (<3% unique mapped reads) were excluded from the analysis based on the results of analysis by principal components analysis (PCA), leaving n=4 females per genotype (*ASNS*$^{+/+}$ and *ASNS*$^{-/-}$). Trimmed reads were mapped to the human reference genome (hg38) using HISAT2 (v2.1.0) [1] Gene expression levels were quantified using StringTie (v1.3.3b) [1]with gene models (v27) from the GENCODE project. Differentially expressed genes were identified using DESeq2 (v 1.22.1)[2]. PCA of log2 transformed of global expression values was conducted using Qlucore Omics Explorer v3.8 (Qlucore AB, Sweden) and q < 0.1 was used as the significance cut-off criteria.

Supporting References

1. Pertea, M., *et al.* StringTie enables improved reconstruction of a transcriptome from RNA-seq reads. *Nat Biotechnol* **33**, 290-295 (2015).
2. Love, M.I., Huber, W. & Anders, S. Moderated estimation of fold change and dispersion for RNA-seq data with DESeq2. *Genome biology* **15**, 550 (2014).

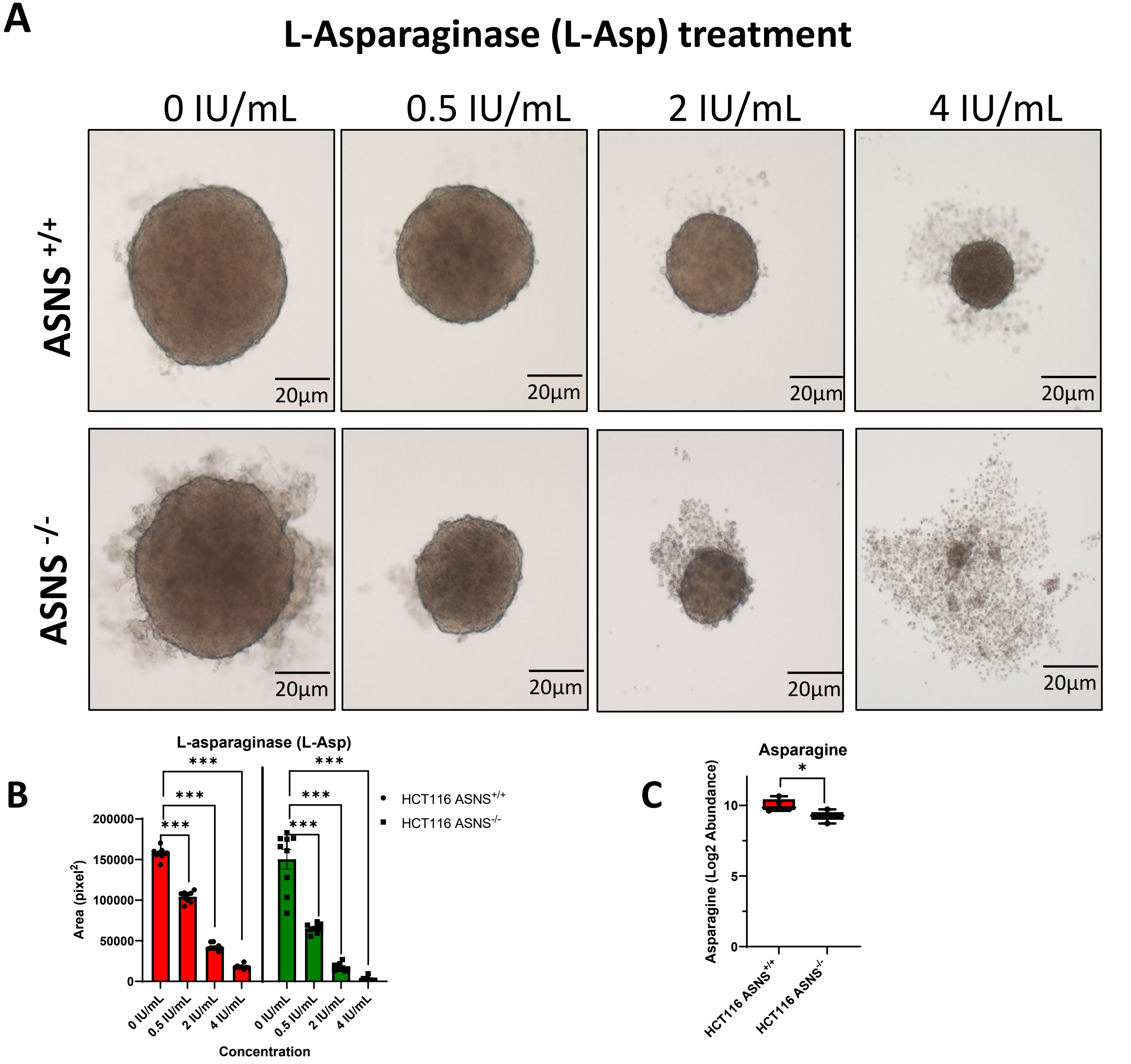

Figure S1: Dose response effects of L-Asparaginase (L-Asp) treatment on HCT116 ASNS $^{+/+}$ and HCT116 ASNS $^{-/-}$ cell lines. (A) L-Asp reduces spheroid formation in the HCT116 *ASNS* lines. Cells were cultured in RPMI with sufficient asparagine and supplemented with 4 mM glutamine to scale up cells before asparaginase treatment. Representative images of spheroids cultured for 7 days after seeding using 4x objective lens in an inverted microscope. 0, 0.5, 2 and 4 IU/mL L-asp provided. (**B**) Quantitation of L-Asp treatment experiment **(C)** Metabolomic analysis showing asparagine levels in HCT116 *ASNS* $^{+/+}$ and HCT116 *ASNS* $^{-/-}$ cell lines. Cells were cultured in RPMI and supplemented with 4 mM glutamine. Individual dot plot represents mean ± SEM with two-way ANOVA used to determine significance in spheroid growth in HCT116 *ASNS* $^{+/+}$ and HCT116 *ASNS* $^{-/-}$ cells treated with L-Asp (**Fig S1B**).* denotes $q < 0.05$ and*** denotes $q < 0.0001$ using Benjamini Hochberg FDR correction method for multiple comparisons.

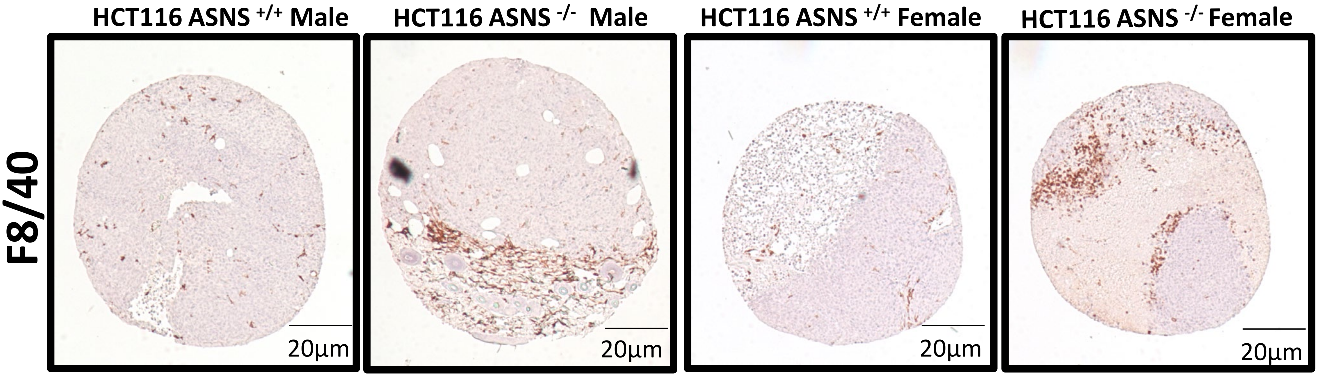

Figure S2: Macrophage population in HCT116 xenografts

Immunohistochemistry analysis of F4/80, a macrophage-selective marker in the HCT116 xenografts. Representative samples (n=5/group) were selected from the TMA cores for F4/80 immunohistochemical staining

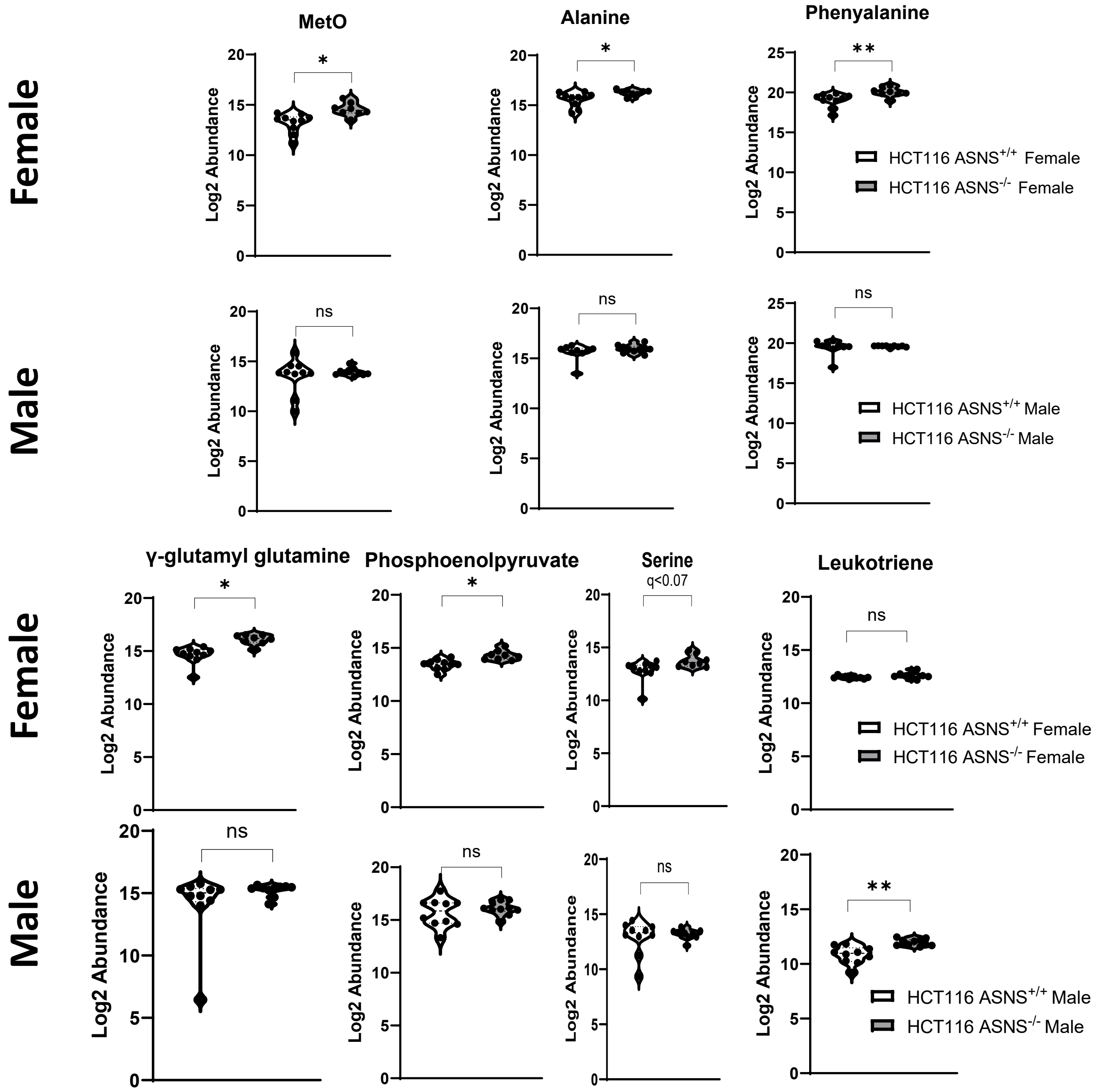

Figure S3: Sex differences in amino acid metabolism and lipid biosynthesis in tumor metabolome. Relative abundance of methionine sulfoxide (MetO), alanine, phenylalanine in the HCT116 *ASNS* $^{+/+}$ and *ASNS* $^{-/-}$ tumors (female and male, top two rows). Relative abundance of gamma-glutamylglutamine, phosphoenolpyruvate, serine and leukotriene B4 (LTB4) in the HCT116 *ASNS* $^{+/+}$ and HCT116 *ASNS* $^{-/-}$ tumors (female and male, bottom two rows). Violin plots showing Log2 abundance of metabolite levels, generated using Graphpad prism. Unpaired student t-test was used to compare mean differences between two groups. Statistical significance was determined using p values adjusted for false discovery rates (FDR). * $q < 0.05$, ** $q < 0.01$, ns = not significant. HCT116 *ASNS* $^{+/+}$ female n = 10, HCT116 *ASNS* $^{-/-}$ female n = 8, HCT116 *ASNS* $^{+/+}$ male n = 10, HCT116 *ASNS* $^{-/-}$ male n= 10.

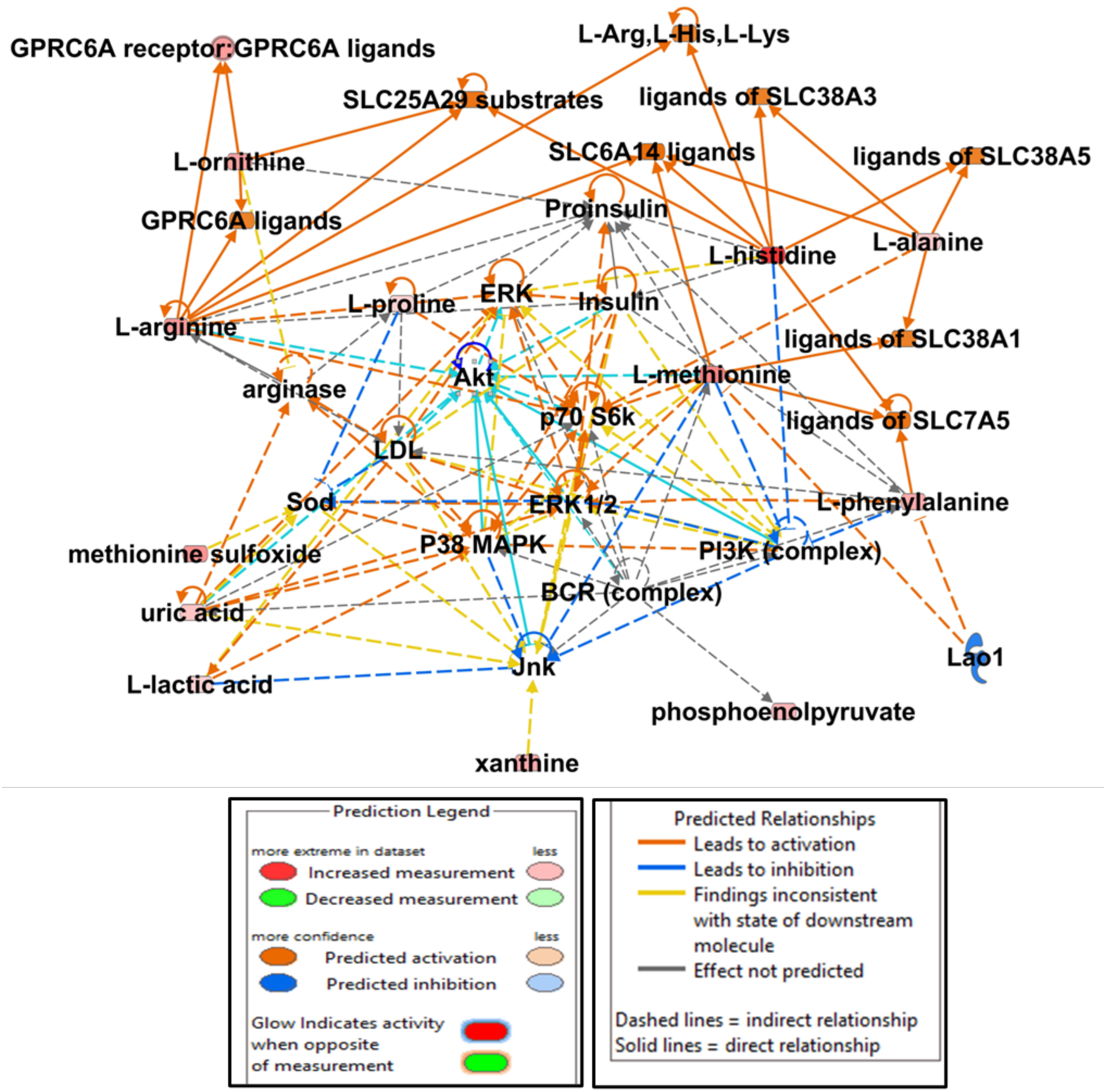

Figure S4: Network analysis of metabolites detected in HCT116 *ASNS* tumor xenograft (female mice, study 2). Network analysis was conducted with Ingenuity Pathway Analysis software on 36 tumor metabolites that were significantly altered in the HCT116 *ASNS* $^{-/-}$ female tumors (n=8) compared to HCT116 *ASNS* $^{+/+}$ female tumors (n=10) from study 2. Significantly increased metabolites are highlighted in red. Network score = 33. The score represents the negative exponent of the right-tailed Fisher's exact test. Statistical significance was determined using q < 0.05.

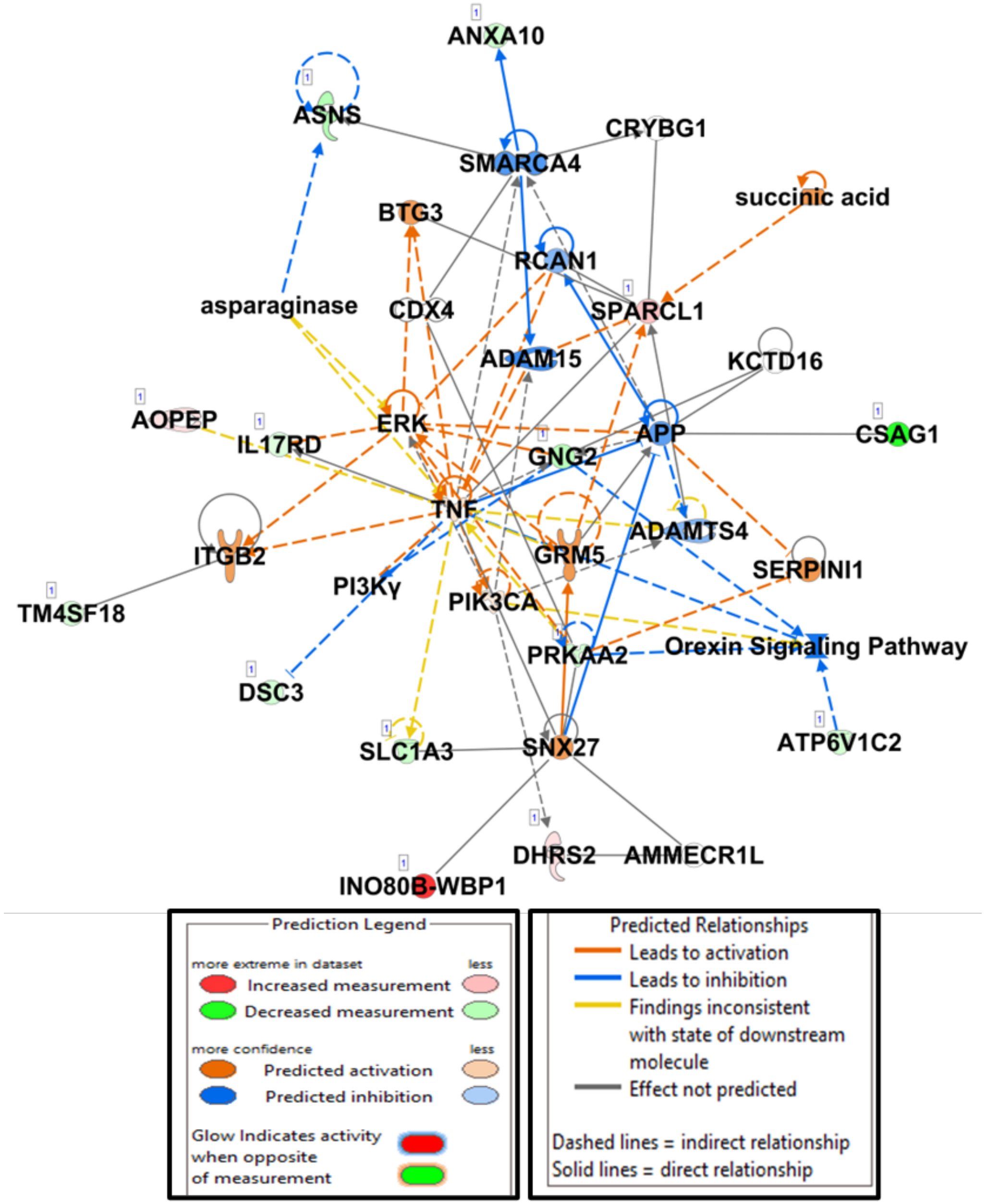

Figure S5: Network analysis of significantly altered genes using IPA (Study 1). ERK and TNF signaling were predicted to be activated by *ASNS* deletion in the female tumor bearing R2G2 mice. Network analysis using the top differentially expressed genes from RNA-Seq data in study 1. Molecules in green indicate significantly downregulated genes and molecules in red indicate significantly upregulation. . Network score = 45. The score represents the negative exponent of the right-tailed Fisher's exact test *p*-value

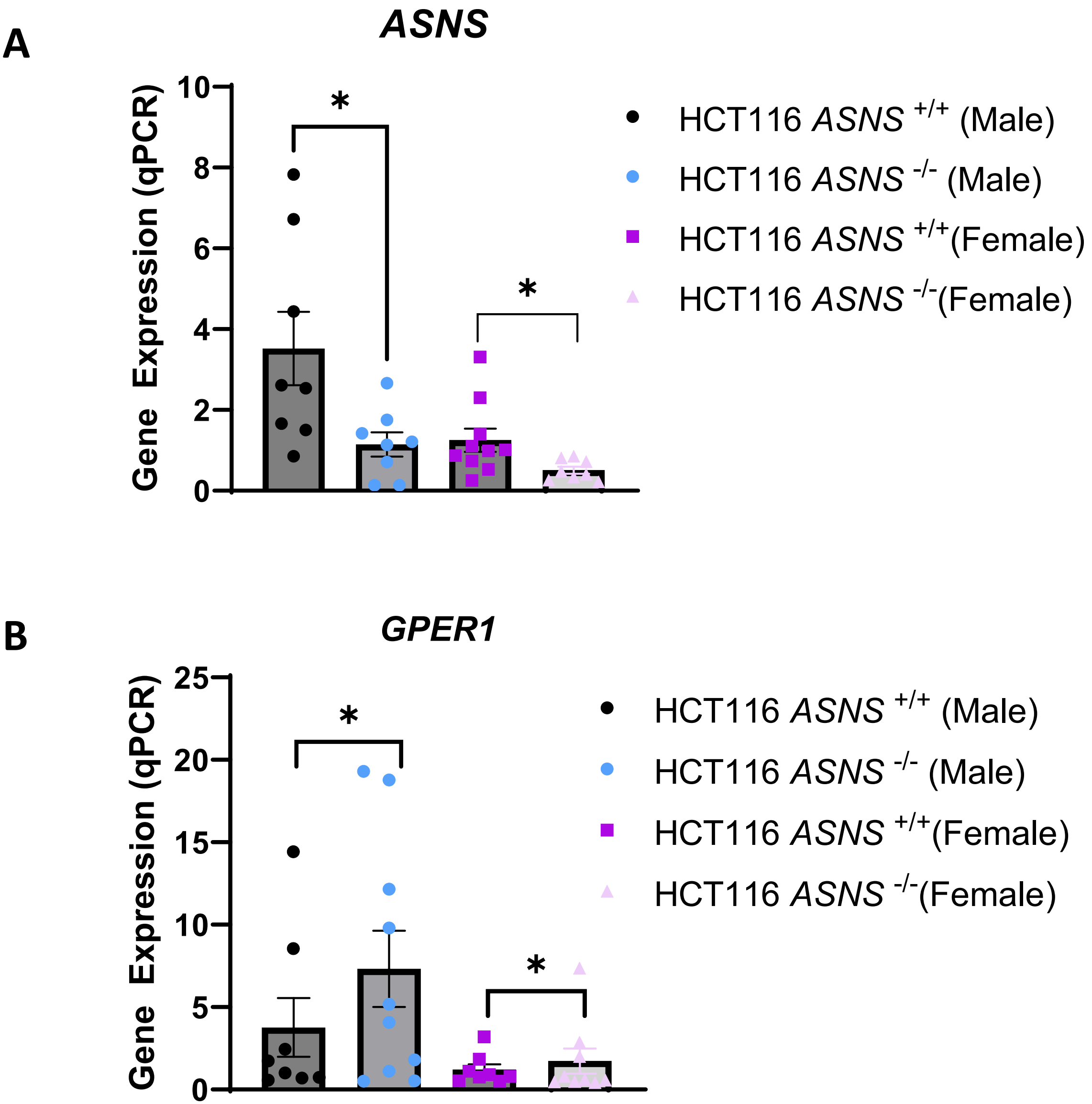

Figure S6: *ASNS* and *GPER1* expression in HCT116 xenografts. Gene expression analysis using real time quantitative polymerase chain reaction for *ASNS* and *GPER1.* (A) ASNS mRNA levels in HCT116 tumor xenografts from male and female R2G2 mice (B) GPER1 mRNA levels in HCT116 tumor xenografts from male and female R2G2 mice. Primer sequences for real-time polymerase chain reaction are listed under Methods. *n=8-10 mice per group.* Individual scattered dot plot represents relative fold change of *ASNS* and *GPER1.* Data are mean ± SEM. Statistical significance indicated by *q < 0.05 using two-way ANOVA with FDR correction for multiple comparison.
